# Design and evaluation of Raman reporters for the Raman-silent region

**DOI:** 10.7150/ntno.58965

**Published:** 2022-01-01

**Authors:** Konstantinos Plakas, Lauren E. Rosch, Michael D. Clark, Shukree Adbul-Rashed, Travis M. Shaffer, Stefan Harmsen, Sanjiv S. Gambhir, Michael R. Detty

**Affiliations:** 1 Department of Chemistry, University at Buffalo, The State University of New York, Buffalo, NY, USA.; 2 Molecular Imaging Program at Stanford University (MIPS), Stanford University School of Medicine, Stanford, CA, USA.; 3 Department of Radiology, Stanford University School of Medicine, Stanford, CA, USA.; 4 Department of Radiology, Perelman School of Medicine, University of Pennsylvania, PA, USA.; 5 Department of Bioengineering, Stanford University School of Medicine, Stanford, CA, USA.; 6 Department of Material Science & Engineering, Stanford University School of Engineering, Stanford, CA, USA.

**Keywords:** Nanotag, surface-enhanced Raman scattering, Raman-silent region, triple bonds

## Abstract

*Rationale:* Surface enhanced Raman scattering (SERS) is proving to be a useful tool for biomedical imaging. However, this imaging technique can suffer from poor signal-to-noise ratio, as the complexity of biological tissues can lead to overlapping of Raman bands from tissues and the Raman reporter molecule utilized. *Methods:* Herein we describe the synthesis of triple bond containing Raman reporters that scatter light in the biological silent window, between 1750 cm^-1^ and 2750 cm^-1^. *Results:* Our SERS nanoprobes are comprised of uniquely designed Raman reporters containing either alkyne- or cyano-functional groups, enabling them to be readily distinguished from background biological tissue. *Conclusion:* We identify promising candidates that eventually can be moved forward as Raman reporters in SERS nanoparticles for highly specific contrast-enhanced Raman-based disease or analyte detection in biological applications.

## Introduction

Surface-enhanced Raman scattering (SERS) has emerged as a highly promising bioanalytical and biomedical imaging platform. SERS is based on amplification of the Raman scattering cross-section of analytes adsorbed on noble-metal (*e.g.,* gold) nanoparticle surfaces 1. Furthermore, the fingerprint-like Raman spectra, which are a reflection of the vibrational transitions of the analyte, enable tremendous multiplex capabilities - simultaneous detection of up to 10 different SERS nanotags has been described in living subjects 2. As such, SERS has not only been used to improve the sensitivity of (multi)analyte detection in bioanalytical assays 3-5, but to generate highly sensitive (targeted) Raman imaging probes for early and comprehensive disease detection *in vivo* 6-8.

To achieve the reported femto- to attomolar limits of detection of these reporter probes, analytes (or Raman reporters) that are resonant with the excitation laser were adsorbed on gold nanoparticles. Typically, these reporters are cyanine- or pyrylium-dyes because the electronic transitions of these dyes can be tuned to match with excitation wavelength of the widely used 785-nm lasers. However, since the Raman fingerprints of cyanine-dyes are complex, and, the fingerprints of pyrylium dyes demonstrate strong overlaps, this limits the multiplexing capabilities of SERS nanoprobes generated from (resonant) Raman reporters of these particular dye classes 9-12. In order to expand the multiplexing capabilities of these classes of dyes and avoid spectral interferences, we sought to optimize the pyrylium dye structures for performance in the Raman silent region (1750-2750 cm^-1^) - a spectral window where Raman bands from biological molecules and tissues are minimized 13, 14 as only Raman bands from triple bond (*e.g.*, alkyne, cyanide, nitrile, *etc*.) vibrational modes appear 15, 16. Since the Raman shift is relative to the energy of the excitation source, this Raman-silent window corresponds to a wavelength range of about 720-775 nm when the excitation wavelength is 638-nm and to a range of 910-1000 nm for a 785-nm excitation source.

**Scheme 1:**
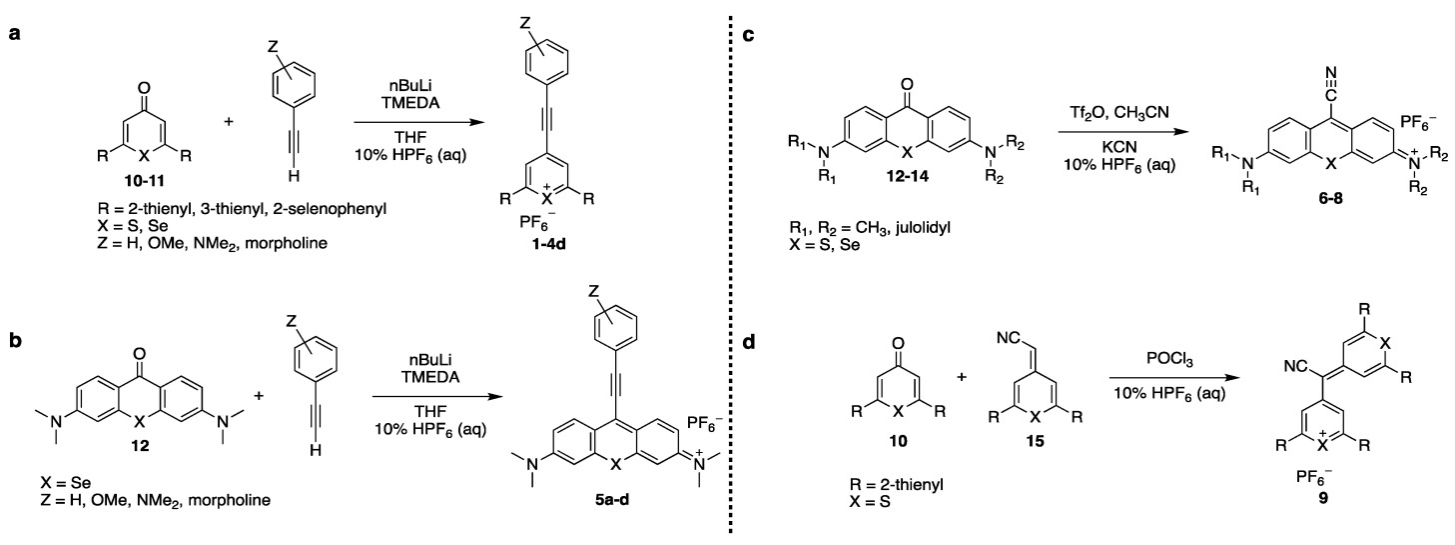
General Synthesis of Raman Reporters.

Here, we modified the pyrylium-scaffold to incorporate alkyne functionality to produce Raman reporters for the Raman silent region. We adapted our previously reported pyrylium dye synthesis procedure 17, which is based on reaction of 4H-chalcogenopyranones with Grignard reagents to incorporate terminal alkynes (**Scheme 1**). In a similar manner, we adapted existing synthesis protocols of xanthone-based chromophores 18, 19 to incorporate alkyne- and cyano-functionality 20. The newly established library of triple-bond containing pyrylium- and xanthylium dyes were subsequently evaluated for use as Raman reporters for the Raman silent region. Here, we report on the identification of several rationally designed Raman reporters that enable multiplexed SERS nanoparticle-based Raman imaging in the Raman silent region.

## Methods

### Materials

All chemicals were obtained from Sigma Aldrich (St. Louis, MO) and were of the highest purity.

### General Synthesis of 4-ethynylphenyl substituted pyrylium or xanthylium dyes (1-5d)

A flame-dried flask fit with a condenser under nitrogen was charged with a substituted phenylacetylene derivative (0.830 mmol), tetramethylenediamine (TMEDA) (0.724 mmol), and anhydrous tetrahydrofuran (THF) (3 mL) and cooled to -78 °C. To this solution, *n-*butyl lithium (0.724 mmol) was added dropwise, and allowed to stir for 15 min at -78 °C, and was subsequently warmed to ambient and stirred for 30 min. This flask was subsequently cooled down to -78 °C. In a separate flame-dried flask under nitrogen, the parent xanthone or pyranone (0.361 mmol) was dissolved in THF (3 mL). The pyranone was transferred to the first flask via cannula. The resulting mixture was allowed to stir at -78 °C for 15 min, before being heated to 50 °C for 15 min. This was subsequently cooled down to ambient temperature. The reaction mixture was then poured into 10% hexafluorophosphoric acid (10 mL). The mixture was filtered, the collected residue was dissolved in methylene chloride (CH_2_Cl_2_) and dried with sodium sulfate. This was filtered, and the filtrate concentrated. The crude product was purified by a traditional recrystallization from boiling CH_3_CN and slowly cooled to yield the desired product.

### General Synthesis of Xanthylium-Cyano-Containing Dye (6-8)

A flame-dried flask under nitrogen was charged with the parent xanthone (0.168 mmol), and CH_3_CN (8 mL). To this solution, trifluoromethanesulfonic anhydride (0.184 mmol) was added dropwise. After 30 min, KCN (0.838 mmol) was added. After 3 h, the reaction mixture was poured into a 10% solution of hexafluorophosphoric acid (30 mL). After stirring overnight, the mixture was filtered, and washed with water and ether (Et_2_O). The collected residue was dissolved in CH_2_Cl_2_ and dried with sodium sulfate. This was filtered, and the filtrate concentrated. The crude product was purified by a two-solvent recrystallization from CH_2_Cl_2_/Et_2_O to yield the desired chromophore.

### General Synthesis of Pyrylium-Cyano Dye (9)

A flame-dried flask fit with a condenser under nitrogen was charged with the parent pyranone (0.334 mmol) exocycliccyanomethylidene derivative (0.367 mmol), and phosphorus oxychloride (0.33 mL). The reaction mixture was heated at reflux for 3 h. The mixture was then cooled to ambient temperature and poured into a 10% hexafluorophosphoric acid solution (20 mL). The organic layer was extracted with methylene chloride (6 15 mL). The combined organic extracts were dried over sodium sulfate, filtered, and concentrated in vacuo. The residue was crystallized from acetonitrile (CH_3_CN). The solid was washed with hot portions of CH_3_CN. The filtrate was concentrated, and the residual solid was purified by a traditional recrystallization from boiling CH_3_CN and slowly cooled to yield the desired chromophore.

### Raman spectroscopy

All dyes were dissolved in dimethyl sulfoxide (DMSO) at a concentration of 30 mM and added to 100 L/well 1.0 nM 60-nm gold nanoparticles in water to yield a final dye concentration of 300 M. After 1-min equilibration, surface-enhanced (resonance) Raman scattering spectra were acquired on a Horiba XplorRA+ Confocal Raman system using 638-nm and 785-nm excitation lasers operating at 638 nm (30 mW) and 785 nm (80 mW) using a 1s acquisition time and operating at 1% laser power.

## Results and Discussion

### Raman reporter synthesis

The triple bond containing pyrylium (**1-4, 9**) or xanthylium dyes (**5-8**) were synthesized according to **Scheme 1** (See Supplemental Information for detailed synthetic schemes). Ethynylphenyl-substituted pyrylium dyes (**1-4d**) were synthesized by terminally deprotonating the appropriate 4-ethynylphenyl derivative using *n*-butyl lithium (nBuLi) and subsequent addition to 4H-chalcogenopyranone (**Scheme 1a**). The hexafluorophosphate salts were obtained by adding the associated alkynyl-substituted pyrylium dyes to 10% hexafluorophosphoric acid solution to yield chromophores **1-4d**. As shown in** Table 1**, apart from the expected contribution of the ethynyl substitution to red-shifting the absorption maxima approximately 50 nm relative to analogous dyes 17, the absorption maxima were further red-shifted over a wide wavelength range by 1) substituting the chalcogen (X) in the pyrylium core with a heavier chalcogen; 2) changing the substituents (R) in the 2- and 6-position of the pyrylium core; and/or 3) by substituting the donor functionality (Z) on the phenyl-group 17. The yields of the 4-ethynylphenyl-substituted pyrylium derivatives (**1-4d**) typically ranged from 15-33% (please see Supplemental Information).

Xanthylium-based chromophores such as rhodamine 800 have been widely applied as Raman reporters 19, 21-24. Inspired by previous work, we therefore synthesized a new class of cyano- and alkyne-containing structures, shown in **Scheme 1b, c** (See Supplemental Information for detailed synthetic schemes), generated from single xanthone-based precursors via nucleophilic attack at the 9-position of the xanthylium core. The incorporation of heavier chalcogen atoms induces sequential bathochromic shifts, favoring resonance enhancement at longer wavelengths. Additionally, constraining the nitrogen atom as seen in moving from the dimethyl amino xanthylium (dye **6**) to the julolidine dyes (dyes **7, 8**) imparts a approximate 10 20-nm bathochromic shift 17. This allows the absorption to be tuned towards the near-infrared region (**Table 2 and 3**). The yields for the 4-ethynylphenyl-substituted and cyano-substituted xanthylium derivatives (**6-8**) ranged from 26-42% and 13-77%, respectively (please see Supplemental Information).

Dye **9** was synthesized by emulating the work performed by VanAllan and coworkers 20, in which cyano-containing pyrylium dyes could be synthesized by allowing cyanoacetic acid to react with pyranones. The chalcogen tripod 25 was adapted in order to engender reporter molecules with higher affinity for typical SERS substrates such as gold or silver nanoparticles. 2,6-di(thiophen-2-yl)-4H-thiopyran-4-one (**10a**) was treated with dimethyl sulfate, thus yielding 4-methoxy-2,6-di(thiophen-2-yl)thiopyrylium hexafluorophosphate (**17**) in 52% yield 26. A subsequent Knoevenagel condensation with cyanoacetic acid yielded 2-(2,6-di(thiophen-2-yl)-4H-thiopyran-4-ylidene)acetonitrile (**15**) in 67% yield, containing both the desired chalcogen tripod and installed cyano group (**See Scheme S1**). As shown in **Scheme 1d**, the pyranone- and exocyclic cyanomethylidene precursors were subsequently condensed in phosphorus oxychloride, thusly forming dye **9** with an absorption maximum of 661 nm.

### Surface-enhanced Raman scattering measurements

To assess and compare the performance of the newly synthesized dyes, the dyes were grouped into four groups: the ethynyl-substituted chalcogenopyrylium dyes (**1-4**); ethynyl-substituted- (**5a-d**); and nitrile-substituted xanthylium dyes (**6-8**), and the cyanopyrylium dye (**9**). The dyes were dissolved in dimethylsulfoxide (DMSO) to yield a concentration of 10 mM and added to a 100-μl dispersion of 60-nm gold nanoparticles (1.0 nM) in water to yield a final dye concentration of 100 μM. We selected 60-nm spherical gold nanoparticles, because those are the most widely-applied size and shape in biomedical applications 6-8, 27-32, and were previously shown to generate the strongest SERS enhancements 33. The SERS spectra were measured in the range of 300-3000 cm^-1^ on a Horiba XploRA+ confocal Raman microscope equipped with 532-nm, 638-nm, and 785-nm excitation lasers. However, here we will only present the SERS spectra after excitation with 638- and 785-nm lasers, because it was found that 532-nm excitation did not produce any appreciable SERS (even at maximum power of 80 mW). Based on theoretical considerations 34, a contribution to the SERS enhancement from the 60-nm gold nanoparticle core was expected, since 532-nm is close to the local surface plasmon resonance of 548 nm of the unmodified 60-nm gold nanoparticles.

**Figure 1:**
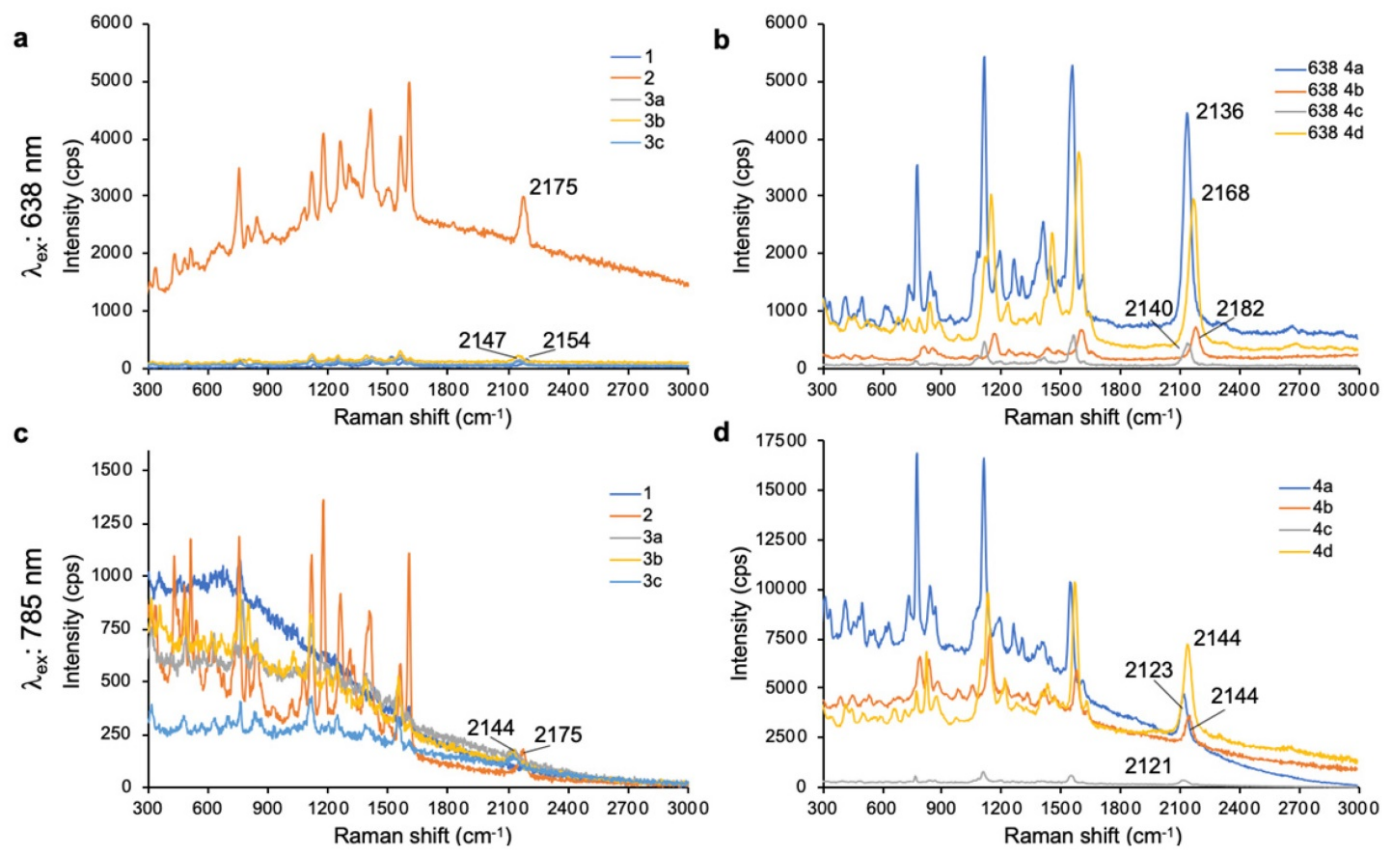
Surface-enhanced Raman scattering spectra of 4-ethynylphenyl substituted pyrylium dyes (**1-4d**). (a) SERS spectra of pyrylium dyes **1-3c** after 638-nm excitation. (b) SERS spectra of pyrylium dyes **4a-d** after 638-nm excitation. (c) SERS spectra of pyrylium dyes **1-3c** after 785-nm excitation. d) SERS spectra of pyrylium dyes **4a-d** after 785-nm excitation.

**Figure 2:**
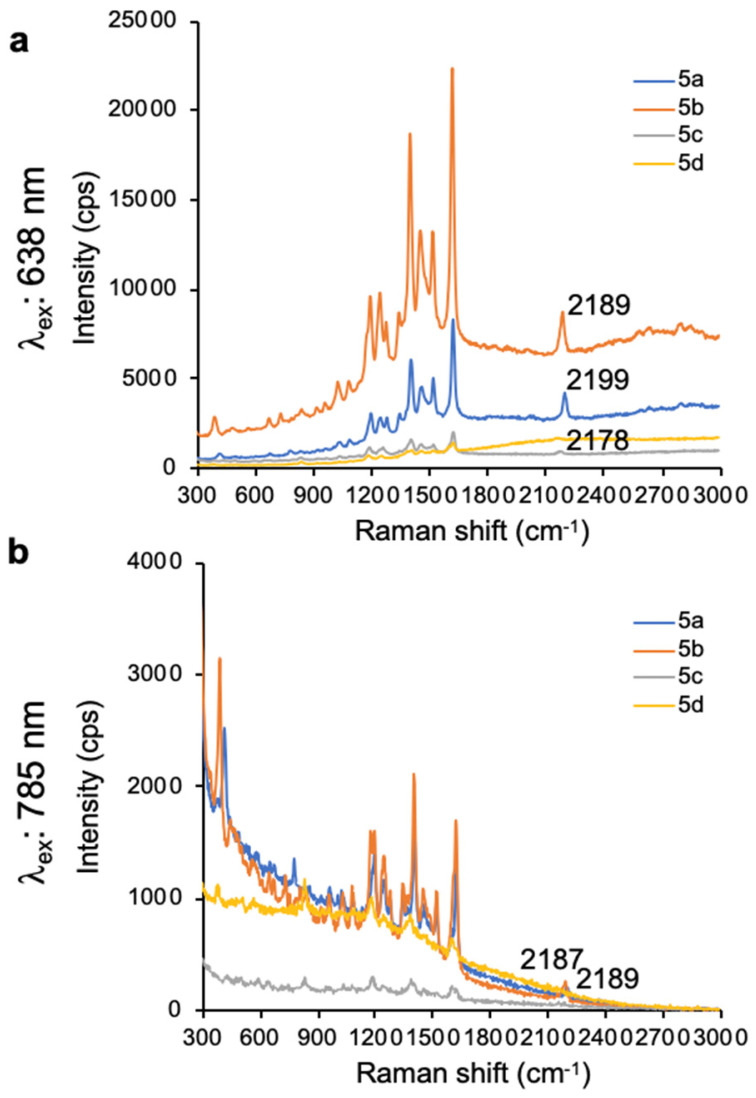
SERS spectra of dyes **5a-d** upon 638-nm (**a**) and 785-nm (**b**) laser excitation.

As shown in **Fig. 1**, 638-and 785-nm excitation of ethynyl-substituted pyrylium dyes **1a-4** led to Raman bands (~2100-2200 cm^-1^) in the Raman silent region (1750-2750 cm^-1^). The Raman shifts of the alkyne match to those previously reported for internal alkynes 16. The dimethylamino-derivatives (**4a** and **4d**) in particular produced Raman bands with high intensities (~3000 cps), relative to the other dyes (<1000 cps). Substitution of the chalcogen atom in the pyrylium ring from sulfur to selenium shifted the Raman band associated with the ring-breathing mode of the aromatic pyrylium ring from 1550- 1560 cm^-1^ to 1570-1590cm^-1^ (depending on the excitation laser wavelength), and also shifted the Raman band associated with the ethynyl stretching mode from 2120-2140 cm^-1^ for the thiopyrylium dyes to 2140-2170 cm^-1^ for the selenopyrylium dyes 16. Furthermore, Yamakoshi *et al.* reported that substituents at the 4-position on the phenyl ring enhanced intensities by extending the π-orbitals in the direction of the alkyne stretching 16. We also found that changing the functionality at the 4-phenyl markedly altered the Raman shift and intensities of the alkyne stretching modes of the chalcogenopyrylium dyes for methoxy- or dimethylamino-substituents relative to a morpholino-substituted phenyl ring or non-substituted phenyl ring (*vide supra*). We further explored this effect of the substituents on the Raman shift of the ethynyl stretching mode in the ethynyl-substituted xanthylium dyes (**5a-d**) that contained different substituents on the phenyl ring adjacent to the ethynyl substituent. We found that the functional groups affected the Raman shift of the ethynyl stretching mode and depending on the substituent induced red- or blue shifts of 10 cm^-1^ relative to the non-substituted phenyl ring (**Fig. 2**). The substituents in the 4-position on the phenyl ring induced a shift in the following order for the alkyne stretching mode from NMe_2_ < morpholine < OMe < H, ranging from 2150 up to 2200 cm^-1^, respectively. However, we did not observe the same effect on intensity of the alkyne stretching mode of the substituted xanthylium dyes, as observed for the chalcogenopyrylium dyes.

**Figure 3:**
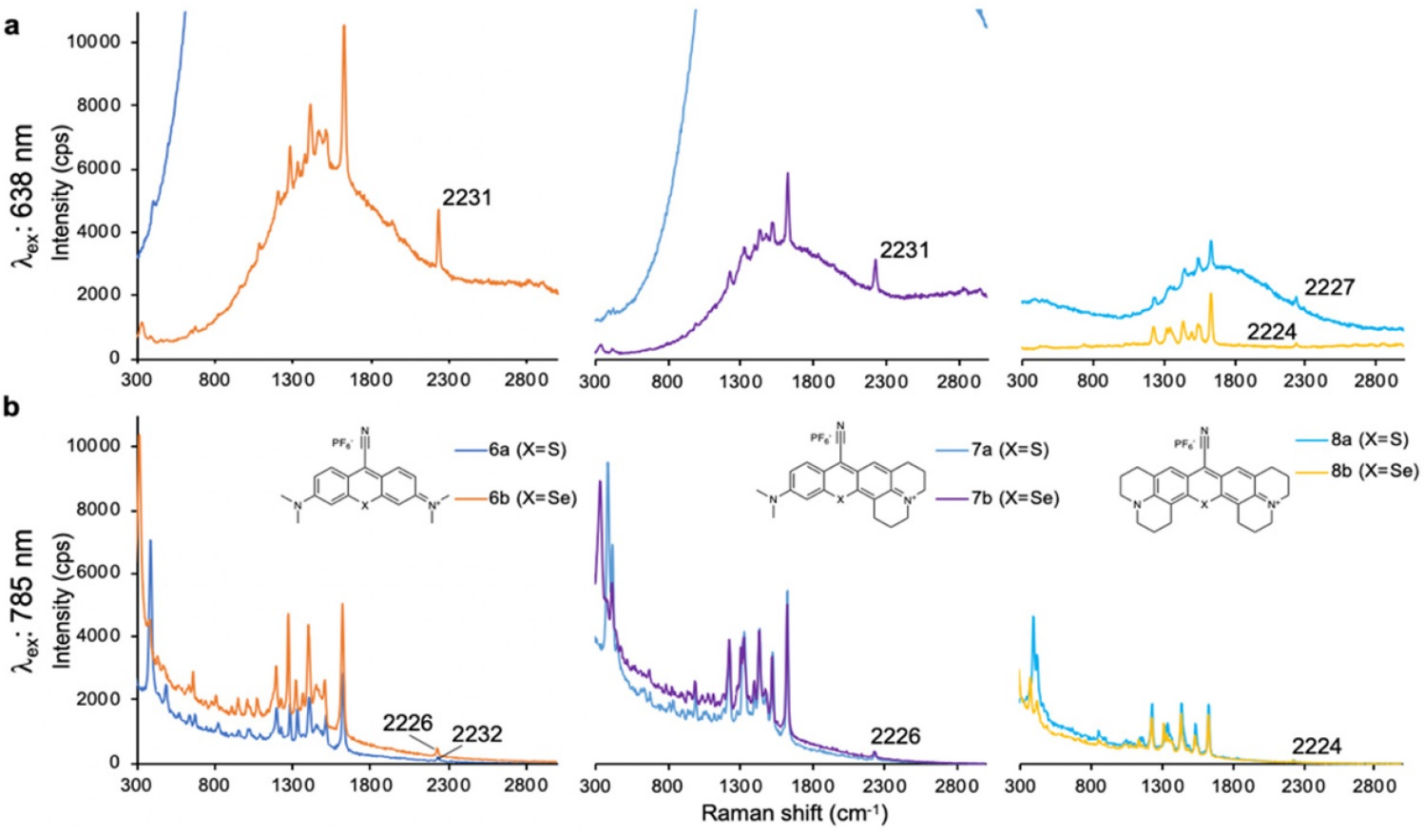
SERS spectra of cyano-substituted thio- (**6a-8a**) and selenoxanthylium (**6b-8b**) dyes upon 638-nm (a) and 785-nm (b) laser excitation

In addition to ethynyl substituted xanthylium dyes, we explored cyano-substituted xanthylium and mono- and bisjulolidyl extended xanthylium dyes as Raman reporters for the Raman silent region (**Table 3**). The two julolidyl moieties impart added rigidity and bathochromic shifts in the wavelength of maximum absorption of the resulting dyes of ~20 nm.^35^ As shown in **Figure 3**, the cyano-substituted selenoxanthylium dyes produced a Raman band in the Raman-silent region around 2230 cm^-1^, while the cyano-substituted thioxanthylium and mono-julolidyl extended thioxanthylium dyes produced strong fluorescence upon 638-nm excitation. Upon 785-nm excitation, which is ~100 nm removed from the excitation maxima of the dyes (**Table 3; Fig. 3**), the fluorescence background of all cyano-substituted xanthylium dyes is markedly reduced and all dyes produce a Raman-band in the Raman-silent region albeit with a relatively weak intensity (*i.e.* ~3-fold reduction) relative to the intensity produced by dye **6b** and **7b** after 638-nm excitation.

**Figure 4:**
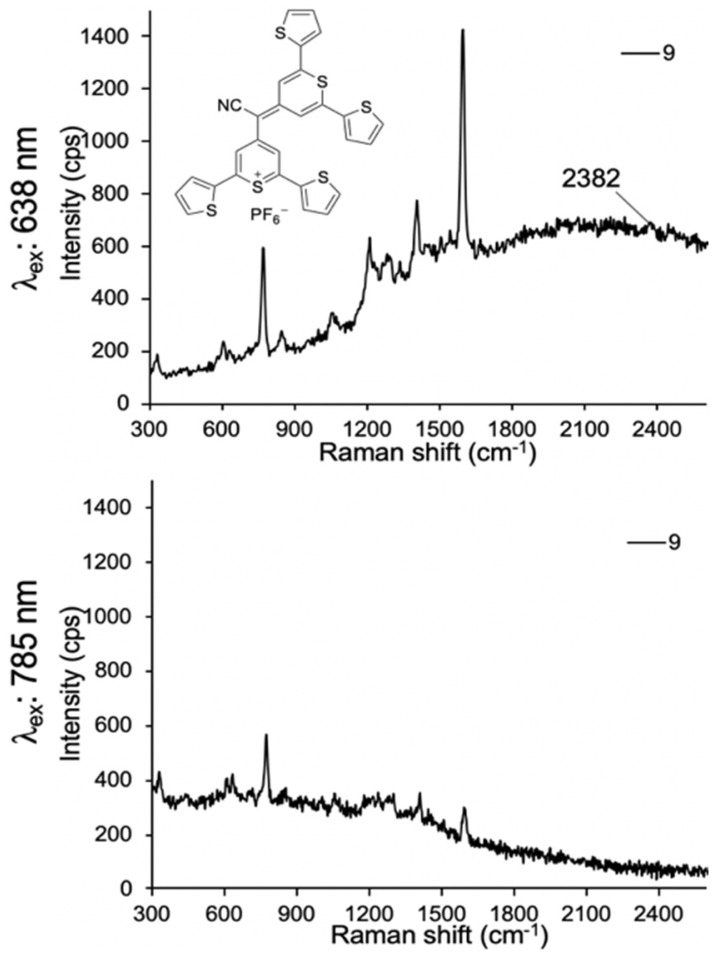
SERS spectra of dye **9** upon 638-nm (a) and 785-nm (b) laser excitation.

Lastly, we evaluated dye **9** where the cyano-functionality was introduced within the conjugated methine system. As shown in **Fig. 4**, dye** 9** produced a weak Raman band at 2383 cm^-1^ after 638-nm laser excitation; a red-shift of an additional 150 cm^-1^ relative to the Raman band produced by cyano-substituted dyes **6-8**. The intensity of the Raman band of -C≡N was too weak to be observed upon 785-nm laser excitation. Possibly, dye 9 is unstable in an aqueous environment and decomposed. We therefore abandoned dye 9.

**Figure 5:**
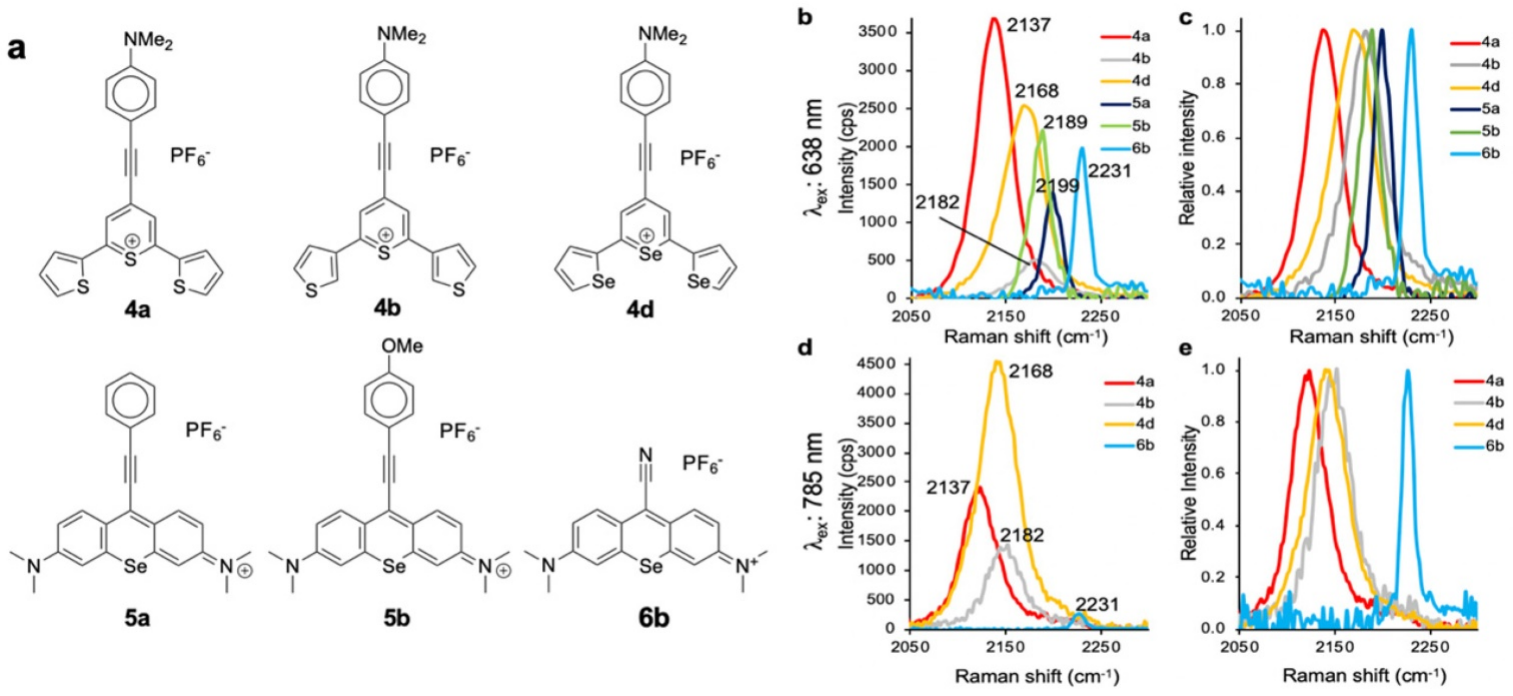
** Raman bands in the Raman-silent region**. **a,** Structures of the selected resonant Raman reporters with the most intense Raman bands in the silent region. **b,** Composite of measured Raman intensity, and **c,** relative Raman intensity upon 638-nm laser excitation. **d,** Composite of measured Raman intensity, and **e,** Relative Raman intensity upon 785-nm laser excitation.

Pyrylium and xanthylium-based dyes represent some of the most sensitive Raman reporters synthesized to date 9, 11, 29. One aim of this work was to generate novel reporters combining the properties that enable high sensitivity, along with functional groups exhibiting vibrational fingerprints in the Raman silent region. As shown in **Fig. 5**, we selected dye** 4a, 4b, 4d, 5a, 5b,** and** 6b** as promising Raman reporters for SERS applications using 638-nm laser excitation (**Fig. 5b-c**) and dyes **4a, 4b, 4d** and **6b** for applications using 785-nm excitation (**Fig. 5d-e**). We excluded dyes **5a** and **5b** as candidates for 785-nm because the intensities of the Raman bands in the Raman-silent region were too low.

The Raman reporters described here should be readily distinguishable from previously synthesized reporters that demonstrated intense pyrylium-breathing modes at or around 1600 cm^-1^. This was accomplished by incorporating both -alkyne and -cyano functional groups into chromophore structures. In the current study, we have demonstrated the synthesis of novel Raman reporter molecules that are capable of displaying resonance enhancement with a 638-nm and 785-nm excitation source and displaying Raman fingerprints in the Raman silent region. It was demonstrated that the di-selenophen-2-yl selenopyrylium dye **4d** exhibited remarkable sensitivity using a 785-nm excitation source. This is likely due to the absorbance of the dye closely overlapping with the excitation source that also has a large extinction coefficient, permitting efficient resonance enhancement to occur. Our work corroborated the findings by Yamakoshi *et al.*^16^ in which the Raman shift displayed by reporter molecules was very sensitive to substituents on the *para*-substituted aromatic rings on the chromophore. Since the stretching frequency is proportional to the square root of the bond strength divided by the reduced mass 19, stronger electron donating substituents may lead to more cumulene-like behavior 36, weakening bond strength and therefore lowering the stretching frequency. This is evident when one compares the phenylacetylene-substituted xanthylium dye **5a** to the analogous anisole derivative dye **5b**. Using the 638-nm excitation source, six Raman bands can be individually identified. This decreased to four when the 785-nm excitation source is used, likely due to minimal resonant enhancement of dyes **5a** and **5b** at this wavelength.

## Conclusions

We have designed, synthesized, and evaluated alkyne- and cyano-substituted chalcogenopyrylium and xanthylium dyes as Raman reporters for the Raman-silent region. We identified 6 candidates that can be used as Raman reporters for SERS applications using 638-nm excitation and 4 candidates for use with a 785-nm excitation source. Moreover, we identified dye **4d** that upon 785-nm laser excitation produced a high-intensity Raman band in the Raman-silent region where Raman background of tissues is minimized. As such, dye **4d** holds great promise as a Raman reporter for a stand-alone SERS-based Raman imaging probe for detection of biomarkers *in vivo* as well.

## Supplementary Material

Supplementary synthesis procedures.

## Figures and Tables

**Table 1:** Structure and absorption maximum of 4-ethynylphenyl pyrylium derivatives.

	Dye	Z	X	R	λ (nm)^*^	log ε
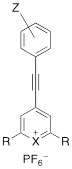	**1**	H	S	thiophen-3-yl	411**	4.48
	**2**	OMe	S	thiophen-2-yl	525	4.81
	**3a**	morpholine	S	thiophen-2-yl	679/503	4.70/4.40
	**3b**	morpholine	S	thiophen-3-yl	592**	4.54
	**3c**	morpholine	Se	thiophen-2-yl	704/521	4.73/4.45
	**4a**	NMe_2_	S	thiophen-2-yl	709/494	4.86/4.38
	**4b**	NMe_2_	S	thiophen-3-yl	684	4.57
	**4c**	NMe_2_	Se	thiophen-2-yl	742/511	4.76/4.30
	**4d**	NMe_2_	Se	selenophen-2-yl	750/527	4.87/4.42

*Absorption spectra were measured of dye solution in dichloromethane unless otherwise noted. **acetonitrile

**Table 2:** Structure and absorption maximum of alkynyl derivatives of selenoxanthylium dyes

	Dye	Z	λ (nm)^*^	log ε
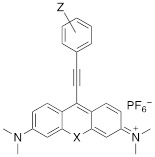	5a	H	632	4.88
	5b	OMe	625	4.98
	5c	morpholine	621	4.97
	5d	NMe_2_	613	5.05

**Table 3:** Structure and absorption maximum of nitrile-derivatives of chalcogenoxanthylium dyes

	Dye	X	R_1_	R_2_	λ (nm)^*^	log ε
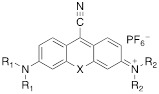	6a	S	CH_3_	CH_3_	662	4.72
	6b	Se	CH_3_	CH_3_	669	4.65
	7a	S	CH_3_	Julolidyl	671	4.91
	7b	Se	CH_3_	Julolidyl	675	4.79
	8a	S	Julolidyl	Julolidyl	687	5.32
	8b	Se	Julolidyl	Julolidyl	692	5.18

## Abbreviations

CH_2_Cl_2_: methylene chloride
CH_3_CN: acetonitrile
DMSO: dimethylsulfoxide
Et_2_O: diethylether
KCN: potassium cyanide
SERS: surface-enhanced Raman scattering
THF: tetrahydrofuran
TMEDA: tetramethylenediamine
