# Supplementary material for: Design and evaluation of Raman reporters for the Raman-silent region
Source: Nanotheranostics. 2022 Jan 1;6(1):1–9. doi: 10.7150/ntno.58965 (PMC8671958; doi:10.7150/ntno.58965)
Supplement: Supplementary file 1 — Supplementary synthesis procedures. [file ntnov06p0001s1.pdf]

SUPPLEMENTARY INFORMATION

Design and evaluation of Raman reporters for the Raman-silent region

Konstantinos Plakas[a*], Lauren E. Rosch[a*], Michael D. Clark[a], Shukree Adbul-Rashed[a],

Travis M. Shaffer[b,c], Stefan Harmsen[b,c,d,#], Sanjiv S. Gambhir[b,c,e,f*,†,], Michael R. Detty[a,*,‡]

Author Affiliations

[a.] Department of Chemistry, University at Buffalo, The State University of New York, Buffalo, NY, USA.

[b.] Molecular Imaging Program at Stanford University (MIPS), Stanford University School of Medicine, Stanford, CA, USA.

[c.] Department of Radiology, Stanford University School of Medicine, Stanford, CA, USA.

[d.] Department of Radiology, Perelman School of Medicine, University of Pennsylvania, PA, USA

[e.] Department of Bioengineering, Stanford University School of Medicine, Stanford, CA, USA.

[f.] Department of Material Science & Engineering, Stanford University School of Engineering, Stanford, CA, USA.

*Authors contributed equally.

†Deceased July 18, 2020

‡Deceased July 1, 2020

#Corresponding author:

Stefan Harmsen, PhD

Email: stefan.harmsen@pennmedicine.upenn.edu

General Experimental Procedures for Chemical Synthesis

All reagents and commercially available starting materials were purchased from commercial sources and used without further purification unless otherwise noted. Dry tetrahydrofuran (THF) was purchased from commercial sources and kept under an inert atmosphere. Other anhydrous solvents were dried over molecular sieves prior to use. Reactions run under anhydrous and/or inert conditions are noted in the procedure. Concentration *in vacuo* was performed on a rotary evaporator. Nuclear Magnetic Resonance (NMR) spectra were recorded on either a 300, 400 or 500 MHz Varian Inova spectrometer for ^{1}H NMR spectroscopy and a 400 MHz Varian Inova spectrometer operating at 100 MHz for ^{13}C NMR spectroscopy. ^{13}C NMR spectra could not be attained for dyes due to solubility issues in common deuterated solvents. Residual solvent signal was used as the internal standard. UV/Vis-near-IR spectra were recorded in glass or quartz cuvettes with a 1-cm pathlength on a Perkin Elmer Lambda 12 spectrophotometer. Melting points were determined with a Mel-Temp melting point apparatus and are uncorrected. Infrared spectra were recorded using a Perkin Elmer Spectrum Two spectrometer using the attenuated total reflectance attachment (ATR). Purity was established to be ≥ 95 % as determined by elemental analyses for C, H, and N for most dye compounds. Experimental values of C, H, and N are within 0.4 % of theoretical values.

Dye precursor compounds **10a-c**[1-5], **12a-b**[6], **13a-b**[7], and **14a-b**[8] were prepared as previously reported.

Synthesis of Novel Raman Reporters

Synthesis of 4-(phenylethynyl)-2,6-di(thien-3-yl)thiopyrylium hexafluorophosphate (1).

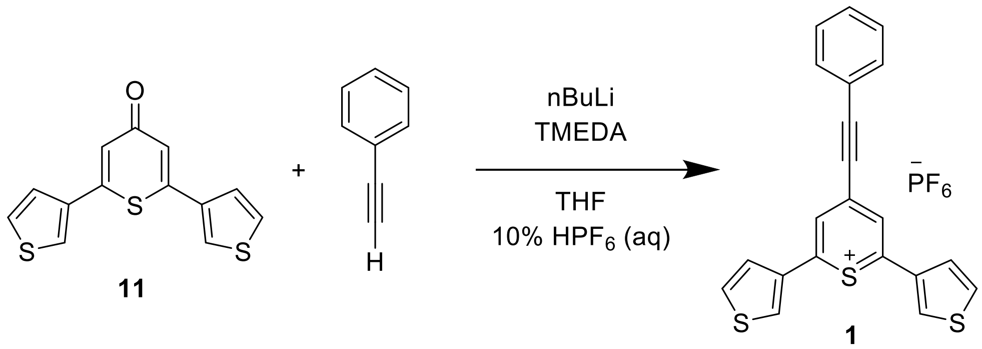

A flame-dried flask fit with a condenser under nitrogen was charged with phenylacetylene (0.085 g, 0.832 mmol), tetramethylenediamine (0.108 mL, 0.724 mmol), and anhydrous tetrahydrofuran (THF) (5 mL) and cooled to -78 °C. To this solution, *n*-butyl lithium (0.63 mL, 0.724 mmol) was added dropwise. In a separate flame-dried flask, 2,6-di(thien-3-yl)-4H-thiopyran-4-one (**11**) (0.100 g, 0.362 mmol) was dissolved in anhydrous THF (5 mL) and cooled to -78 °C. After 15 min, the contents of the second flask were transferred via cannula to the first flask. The reaction mixture was then allowed to warm to ambient temperature, followed by heating at 50 °C for 15 min. The reaction mixture was then cooled to ambient temperature and dumped into a 10 % solution of hexafluorophosphoric acid (30 mL). After 3 h, this was filtered and the crude product was dissolved in CH_2Cl_2 20 mL, dried over sodium sulfate (Na_2SO_4), and concentrated *in vacuo*. The crude product was purified by a traditional recrystallization from boiling CH_3CN and slowly cooled to yield 47.8 mg (26 %) of a dark red crystalline solid, mp >260 °C : ^{1}H NMR [400 MHz, CD_3CN] δ 8.67 (s, 2 H), 8.50 (s, 2 H), 7.79 (m, 6 H), 7.65 (t, 1 H, J = 7.2 Hz), 7.57 (t, 2 H, J = 7.2 Hz); λ_{max} (CH_3CN) 411 nm ($\varepsilon = 3.0 \times 10^4$ M^{-1} cm^{-1}); IR (ATR) 2195.0 cm^{-1} (m, alkyne), 1572.7 cm^{-1} (m, aromatic C=C); HRMS (ESI) *m/z* 361.0168 (calcd for $C_{21}H_{13}S_3$: 361.0173).

Synthesis of 4-((4-methoxyphenyl)ethynyl)-2,6-di(thiophen-2-yl)thiopyrylium hexafluorophosphate (2).

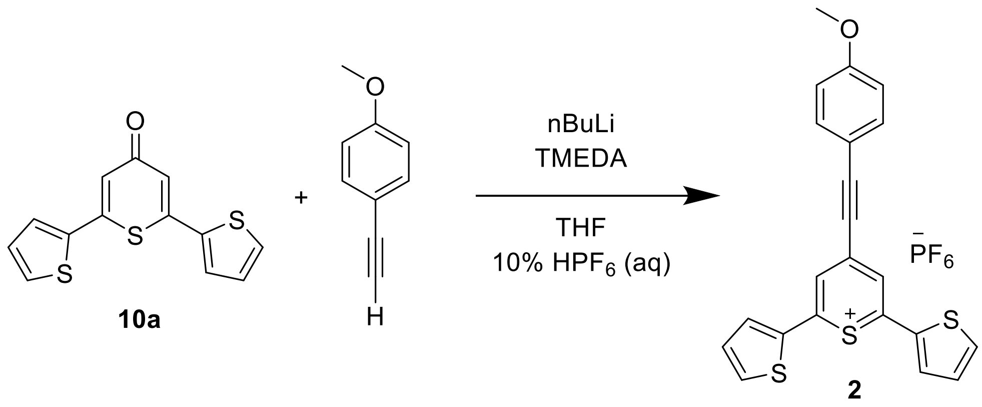

A flame-dried flask fit with a condenser under nitrogen was charged with 4-ethynyl anisole (0.11 mL, 0.8303 mmol), tetramethylenediamine (0.11 mL, 0.724 mmol), and THF (3 mL) and cooled to -78 °C. To this solution, *n*-butyl lithium (0.63 mL, 0.724 mmol) was added dropwise. In a separate flame-dried flask, 2,6-di(thiophen-2-yl)-4H-thiopyran-4-one (**10a**) (100.0 mg, 0.361 mmol) was dissolved in THF (3 mL) and cooled to -78 °C. After 15 min, the contents of the second flask were transferred via cannula, to the first flask. The reaction mixture was then allowed to warm to ambient temperature, followed by heating at 50 °C for 15 min. The reaction mixture was cooled to ambient and poured into a 10 % solution of hexafluorophosphoric acid (20 mL). The mixture was filtered, the collected residue was dissolved in CH_2Cl_2 and dried with sodium sulfate. This was filtered, and the filtrate concentrated. The crude product was purified by a traditional recrystallization from boiling CH_3CN and slowly cooled to yield 52.2 mg (26 %) of a metallic red solid, mp 179-181 °C: ^1H NMR [400 MHz, CD_2Cl_2] δ 8.22 (s, 2 H), 8.04 (d, 2 H, *J* = 3.6), 8.02 (d, 2 H, *J* = 4.8 Hz), 7.78 (d, 2 H, *J* = 8.8 Hz), 7.44-7.42 (m, 2 H), 7.05 (d, 2 H, *J* = 8.8 Hz), 3.92 (s, 3 H,); λ_{max} (CH_2Cl_2) 523 nm ($\varepsilon = 6.50 \times 10^4$ $M^{-1}cm^{-1}$); IR (ATR) 2184.0 cm^{-1} (m, alkyne) 1564.4 cm^{-1} (m, aromatic C=C); HRMS (ESI) *m/z* 391.0281 calcd for $C_{22}H_{15}O_1S_3^+$: 391.0279); Anal calcd for $C_{22}H_{15}F_6OPS_3$: C, 49.25; H, 2.82. Found: C, 49.38; H, 3.02.

Synthesis of 4-((4-morpholinophenyl)ethynyl)-2,6-di(thiophen-2-yl)thiopyrylium hexafluorophosphate (3a).

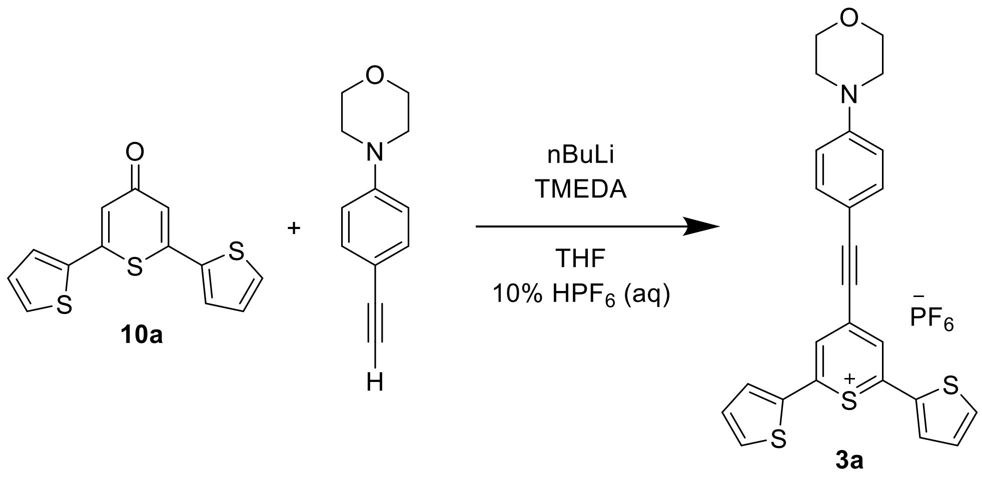

A flame-dried flask fit with a condenser under nitrogen was charged with 4-(4-ethynylphenyl)morpholine (100.0 mg, 0.534 mmol), tetramethylenediamine (69.4 μL, 0.464 mmol), and THF (2.00 mL) and cooled to -78 °C. To this solution, *n-butyl* lithium (0.40 mL, 0.464 mmol) was added dropwise. In a separate flame-dried flask, 2,6-di(thiophen-2-yl)-4H-thiapyran-4-one (**10a**) (64.2 mg, 0.232 mmol) was dissolved in THF (2.00 mL) and cooled to -78 °C. After 15 min, the contents of the second flask were transferred via cannula, to the first flask. The reaction mixture was then allowed to warm to ambient, followed by heating at 50 °C for 15 min. The reaction mixture was cooled to ambient and dumped into a 10 % solution of hexafluorophosphoric acid (10 mL). The mixture was filtered, the collected residue was dissolved in CH_2Cl_2 and dried with sodium sulfate. This was filtered, and the filtrate concentrated. The crude product was purified by a traditional recrystallization from boiling CH_3CN and slowly cooled to yield 60.0 mg (33 %) of a metallic brown solid, mp 199-201 °C: ^{1}H NMR [500 MHz, CD_2Cl_2] δ 8.05 (s, 2 H), 7.98 (d, 2 H, *J* = 5.5 Hz), 7.91 (d, 2 H, *J* = 4.0 Hz), 7.71 (d, 2 H, *J* = 8.5 Hz), 7.41-7.39 (m, 2 H), 6.96 (d, 2 H, *J* = 9.0 Hz), 3.85 (t, 4 H, J = 5.0 Hz), 3.45 (t, 4 H, J = 5.0 Hz); λ_{max} (CH_2Cl_2) 679 nm ($\varepsilon = 5.00 \times 10^4$ $M^{-1}cm^{-1}$), 503 nm ($\varepsilon = 2.04 \times 10^4$ $M^{-1}cm^{-1}$); IR (ATR) 2164.6 cm^{-1} (m, alkyne) 1603.3 cm^{-1} (m, aromatic C=C), 1379.5 cm^{-1} (m, C-N); HRMS (ESI) *m/z* 446.0699 (calcd for $C_{25}H_{20}N_1O_1S_3^+$: 446.0701); Anal calcd for $C_{25}H_{20}F_6NOPS_3$: C, 50.76; H, 3.41; N, 2.37. Found: C, 50.39; H, 3.41; N, 2.46.

Synthesis of 4-((4-morpholinophenyl)ethynyl)-2,6-di(thiophen-3-yl)thiopyrylium hexafluorophosphate (3b).

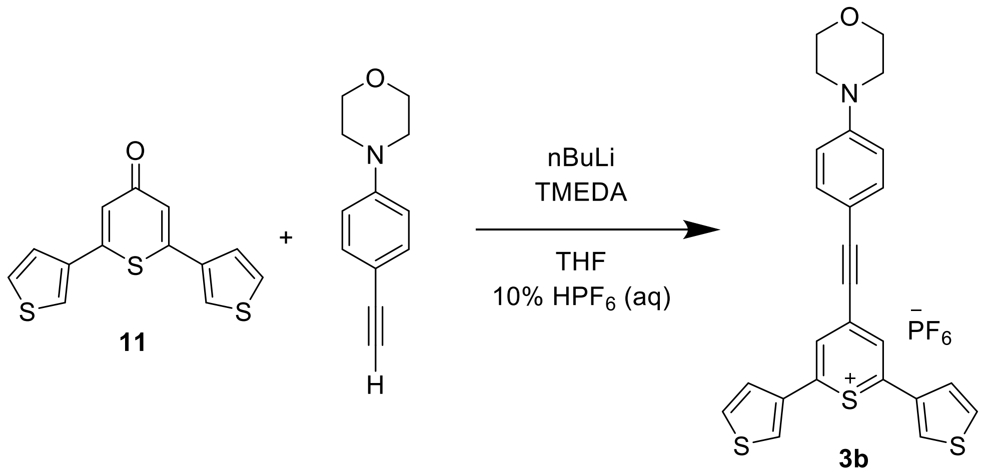

A flame-dried flask fit with a condenser under nitrogen was charged with 4-(4-ethynylphenyl)morpholine (0.149 g, 0.796 mmol), tetramethylenediamine (0.108 mL, 0.724 mmol), and THF (2.5 mL) and cooled to -78 °C. To this solution, *n*-butyl lithium (0.36 mL, 0.724 mmol) was added dropwise. In a separate flame-dried flask, 2,6-di(thien-3-yl)-4*H*-thiopyran-4-one (**11**) (1.00 g, 0.362 mmol) was dissolved in THF (2.5 mL) and cooled to -78 °C. After 15 min, the contents of the second flask were transferred via cannula to the first flask. The reaction mixture was then allowed to warm to ambient temperature, followed by heating at 50 °C for 15 min. The reaction mixture was then cooled to ambient temperature and dumped into a 10 % solution of hexafluorophosphoric acid (30 mL). After 3 h, this was filtered and the crude product was purified by a traditional recrystallization from boiling CH_3CN and slowly cooled to yield 79.9 mg (38 %) of a green solid, mp 240-241 °C: ^{1}H NMR [400 MHz, CD_3CN] δ 8.47 (s, 2 H), 8.41 (s, 2 H), 7.78 (s, 2 H), 7.74 (s, 2 H), 7.66 (d, 2 H, J = 8.8 Hz), 7.02 (d, 2 H, J = 8.4 Hz), 3.78 (s, 4 H), 3.40 (s, 4 H); λ_{max} (CH_3CN) 592 nm ($\varepsilon = 3.5 \times 10^4$ M^{-1} cm^{-1}); IR (ATR) 2171.5 cm^{-1} (m, alkyne), 1567.9 cm^{-1} (m, aromatic C=C), 1363.9 cm^{-1} (m, C-N); HRMS (ESI) *m/z* 446.0706 (calcd for $C_{25}H_{20}NOS_3$: 446.0702).

Synthesis of 4-((4-morpholinophenyl)ethynyl)-2,6-di(thiophen-2-yl)selenopyrylium hexafluorophosphate (3c).

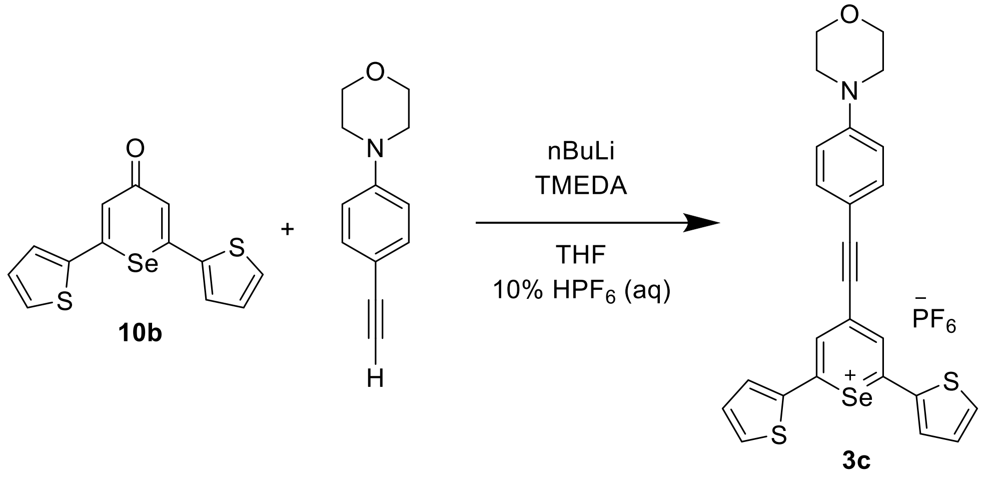

A flame-dried flask fit with a condenser under nitrogen was charged with 4-(4-ethynylphenyl)morpholine (133.1 mg, 0.711 mmol), tetramethylenediamine (92.5 μL, 0.618 mmol), and THF (2.00 mL) and cooled to -78 °C. To this solution, *n-butyl* lithium (0.70 mL, 0.618 mmol) was added dropwise. In a separate flame-dried flask, 2,6-di(thiophen-2-yl)-4H-selenapyran-4-one (**10b**) (100.0 mg, 0.309 mmol) was dissolved in THF (2.00 mL) and cooled to -78 °C. After 15 min, the contents of the second flask were transferred via cannula, to the first flask. The reaction mixture was then allowed to warm to ambient, followed by heating at 50 °C for 15 min. The reaction mixture was cooled to ambient and poured into a 10 % solution of hexafluorophosphoric acid (10 mL). The mixture was filtered, the collected residue was dissolved in CH_2Cl_2 and dried with sodium sulfate. This was filtered, and the filtrate concentrated. The crude product was purified by a traditional recrystallization from boiling CH_3CN and slowly cooled to yield 45.0 mg (23 %) of a metallic brown solid, mp > 260 °C: ^{1}H NMR [500 MHz, CD_2Cl_2] δ 8.07 (s, 2 H), 7.99 (d, 2 H, J = 5.5 Hz), 7.92 (d, 2 H, J = 3.0 Hz), 7.74 (d, 2 H, J = 9.0 Hz), 7.41-7.39 (m, 2 H), 7.05 (d, 2 H, J = 9.0 Hz), 3.85 (t, 4 H, J = 5.0 Hz), 3.45 (t, 4 H, J = 5.0 Hz); λ_{max} (CH_2Cl_2) 704 nm (ε = 5.40 × 10^4 $M^{-1}cm^{-1}$), 521 nm (ε = 2.80 × 10^4 $M^{-1}cm^{-1}$); IR (ATR) 2180.0 cm^{-1} (m, alkyne) 1602.5 cm^{-1} (m, aromatic C=C), 1378.6 cm^{-1} (m, C-N); HRMS (ESI) *m/z* 491.0124 (calcd for $C_{25}H_{20}N_1O_1S_2Se^+$: 491.0130); Anal calcd for $C_{25}H_{20}F_6NOPS_2Se$: C, 47.03; H, 3.16; N, 2.19. Found: C, 46.96; H, 3.26; N, 2.29.

Synthesis of 4-((4-(dimethylamino)phenyl)ethynyl)-2,6-di(thiophen-2-yl)thiopyrylium hexafluorophosphate (4a).

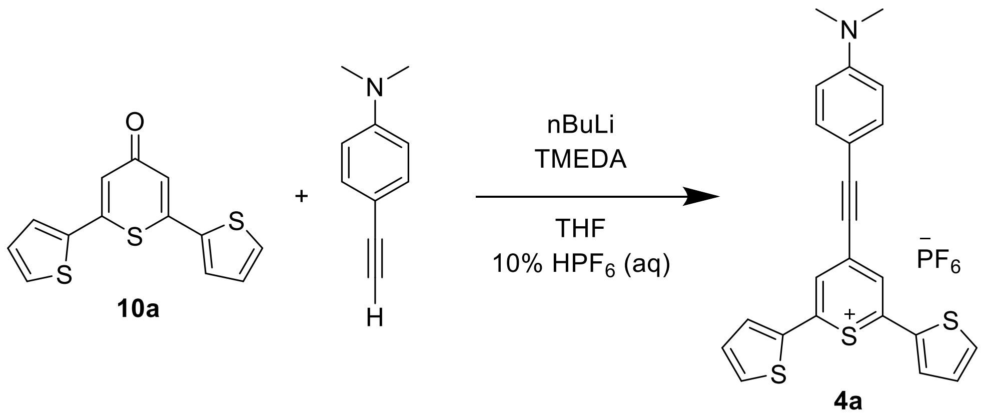

A flame-dried flask fit with a condenser under nitrogen was charged with 4-ethynyl-*N*, *N*-dimethyl aniline (120.6 mg, 0.8303 mmol), tetramethylenediamine (0.11 mL, 0.724 mmol), and THF (3 mL) and cooled to -78 °C. To this solution, *n*-butyl lithium (0.63 mL, 0.724 mmol) was added dropwise. In a separate flame-dried flask, 2,6-di(thiophen-2-yl)-4H-thiopyran-4-one (**10a**) (100.0 mg, 0.361 mmol) was dissolved in THF (3 mL) and cooled to -78 °C. After 15 min, the contents of the second flask were transferred via cannula, to the first flask. The reaction mixture was then allowed to warm to ambient, followed by heating at 50 °C for 15 min. The reaction mixture was cooled to ambient and dumped into a 10 % solution of hexafluorophosphoric acid (10 mL). The mixture was filtered, the collected residue was dissolved in CH_2Cl_2 and dried with sodium sulfate. This was filtered, and the filtrate concentrated. The crude product was purified by a traditional recrystallization from boiling CH_3CN and slowly cooled to yield 52.1 mg (26 %) of a metallic green solid, mp 218-220 °C: ^{1}H NMR [500 MHz, CD_2Cl_2] δ 8.02 (s, 2 H), 7.92 (m, 4 H), 7.68 (d, 2 H, *J* = 8.5 Hz), 7.38 (s, 2 H), 6.82 (d, 2 H, *J* = 4.5 Hz), 3.19 (s, 6 H,); λ_{max} (CH_2Cl_2) 709 nm (ε = 7.20 × 10^4 $M^{-1}cm^{-1}$), 494 nm (ε = 2.40 × 10^4 $M^{-1}cm^{-1}$); IR (ATR) 2121.2 cm^{-1} (m, alkyne) 1600.4 cm^{-1} (m, aromatic C=C), 1387.3 cm^{-1} (m, C-N); HRMS (ESI) *m/z* 404.0597 (calcd for $C_{23}H_{18}N_1S_3^+$: 404.0595); Anal calcd for $C_{23}H_{18}F_6NPS_3$: C, 50.27; H, 3.30; N, 2.55. Found: C, 50.40; H, 3.47; N, 2.72.

Synthesis of 4-((4-(dimethylamino)phenyl)ethynyl)-2,6-di(thiophen-3-yl)thiopyrylium hexafluorophosphate (4b).

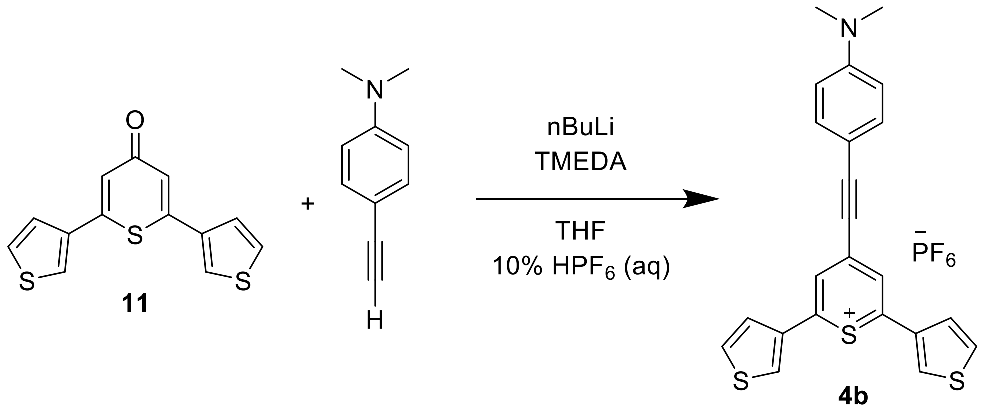

A flame-dried flask fit with a condenser under nitrogen was charged with 4-ethynyl-*N*,*N*-dimethylaniline (0.121 g, 0.832 mmol), tetramethylenediamine (0.108 mL, 0.724 mmol), and THF (2.5 mL) and cooled to -78 °C. To this solution, *n*-butyl lithium (0.38 mL, 0.724 mmol) was added dropwise. In a separate flame-dried flask, 2,6-di(thien-3-yl)-4*H*-thiopyran-4-one (**11**) (0.100 g, 0.362 mmol) was dissolved in THF (2.5 mL) and cooled to -78 °C. After 15 min, the contents of the second flask were transferred, via cannula, to the first flask. The reaction mixture was then allowed to warm to ambient temperature, followed by heating at 50 °C for 15 min. The reaction mixture was then cooled to ambient temperature and dumped into a 10 % solution of hexafluorophosphoric acid (30 mL). After 3 h, this was filtered and the crude product was purified by a traditional recrystallization from boiling CH_3CN and slowly cooled to yield 54.0 mg (27 %) of a green solid, mp >260 °C: ^1H NMR [400 MHz, CD_3CN] δ 8.39 (d, 4 H, J = 11.2 Hz), 7.77 (t, 2 H, J = 4.8 Hz), 7.71 (d, 2 H, J = 5.2 Hz), 7.64 (d, 2 H, J = 8.8 Hz), 6.89 (d, 2 H, J = 8.8 Hz), 3.12 (s, 6 H); λ_{max} (CH_2Cl_2) 684 nm ($\varepsilon = 3.7 \times 10^4$ M^{-1} cm^{-1}); IR (ATR) 2141.0 cm^{-1} (m, alkyne), 1556.5 cm^{-1} (m, aromatic C=C), 1372.7 cm^{-1} (m, C-N); HRMS (ESI) *m/z* 404.0589 (calcd for $C_{23}H_{18}NS_3$: 404.0596).

Synthesis of 4-((4-(dimethylamino)phenyl)ethynyl)-2,6-di(thiophen-2-yl)selenopyrylium hexafluorophosphate (4c).

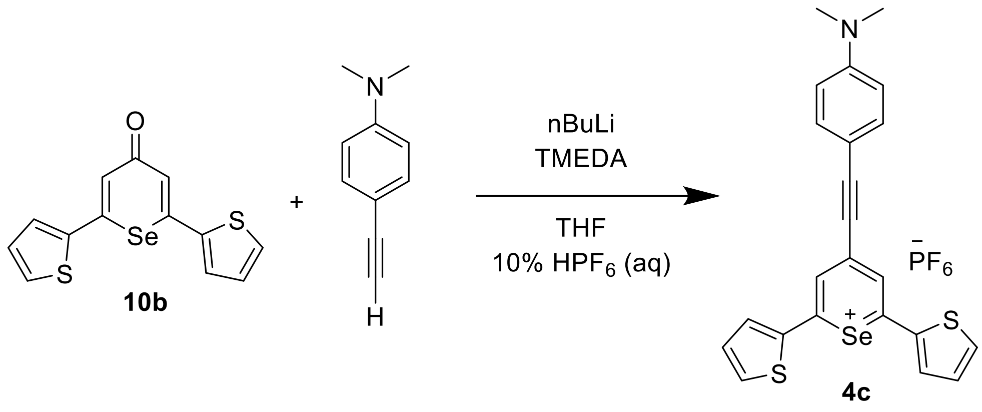

A flame-dried flask fit with a condenser under nitrogen was charged with 4-ethynyl-*N*, *N*-dimethyl aniline (103.2 mg, 0.711 mmol), tetramethylenediamine (92.5 µL, 0.618 mmol), and THF (2.60 mL) and cooled to -78 °C. To this solution, *n*-butyl lithium (0.70 mL, 0.618 mmol) was added dropwise. In a separate flame-dried flask, 2,6-di(thiophen-2-yl)-4H-selenapyran-4-one (**10b**) (100.0 mg, 0.309 mmol) was dissolved in THF (2.60 mL) and cooled to -78 °C. After 15 min, the contents of the second flask were transferred via cannula, to the first flask. The reaction mixture was then allowed to warm to ambient, followed by heating at 50 °C for 15 min. The reaction mixture was cooled to ambient and dumped into a 10% solution of hexafluorophosphoric acid (20 mL). The mixture was filtered, the collected residue was dissolved in CH_2Cl_2 and dried with sodium sulfate. This was filtered, and the filtrate concentrated. The crude product was purified by a traditional recrystallization from boiling CH_3CN and slowly cooled to yield 60.0 mg (33 %) of a metallic brown solid, mp 172-174 °C: ^{1}H NMR [400 MHz, CD_2Cl_2] δ 7.98 (s, 2 H), 7.92 (d, 2 H, *J* = 5.2 Hz), 7.84 (d, 2 H, *J* = 2.8 Hz), 7.68 (d, 2 H, *J* = 8.8 Hz), 7.38-7.35 (m, 2 H,) 6.83 (d, 2 H, *J* = 8.8 Hz), 3.18 (s, 6 H,); λ_{max} (CH_2Cl_2) 742 nm ($\varepsilon = 5.80 \times 10^4$ $M^{-1}cm^{-1}$), 511 nm ($\varepsilon = 2.00 \times 10^4$ $M^{-1}cm^{-1}$); IR (ATR) 2120.3 cm^{-1} (m, alkyne) 1604.5 cm^{-1} (m, aromatic C=C), 1397.1 cm^{-1} (m, C-N); HRMS (ESI) *m/z* 452.0017 (calcd for $C_{23}H_{18}N_1S_2Se^+$: 452.0025); Anal calcd for $C_{23}H_{18}F_6NPS_2Se$: C, 46.32; H, 3.04; N, 2.35. Found: C, 46.46; H, 3.22; N, 2.55.

Synthesis of 4-((4-(dimethylamino)phenyl)ethynyl)-2,6-di(selenophen-2-yl)selenopyrylium hexafluorophosphate (4d).

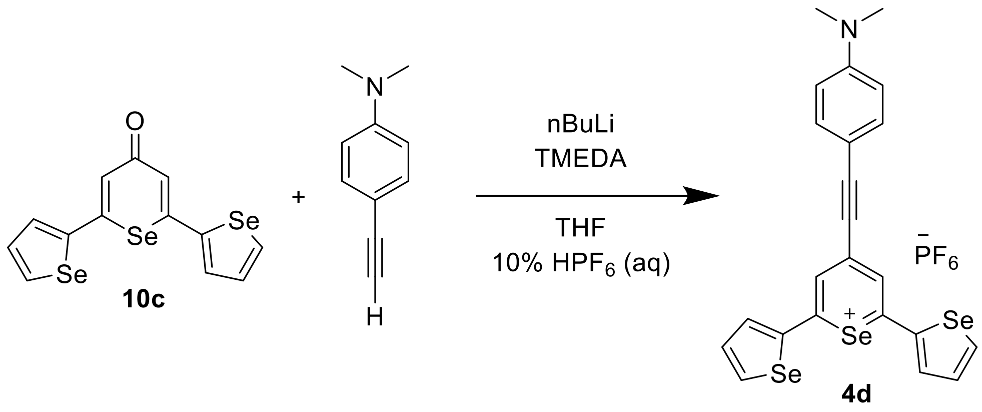

A flame-dried flask fit with a condenser under nitrogen was charged with 4-ethynyl-*N*, *N*-dimethyl aniline (160.0 mg, 1.102 mmol), tetramethylenediamine (0.14 mL, 0.960 mmol), and THF (4.00 mL) and cooled to -78 °C. To this solution, *n-butyl* lithium (1.40 mL, 0.960 mmol) was added dropwise. In a separate flame-dried flask, 2,6-di(selenophen-2-yl)-4H-selenapyran-4-one (**10c**) (200.0 mg, 0.480 mmol) was dissolved in THF (4.00 mL) and cooled to -78 °C. After 15 min, the contents of the second flask were transferred via cannula, to the first flask. The reaction mixture was then allowed to warm to ambient, followed by heating at 50 °C for 15 min. The reaction mixture was cooled to ambient and dumped into a 10 % solution of hexafluorophosphoric acid (30 mL). The mixture was filtered, the collected residue was dissolved in CH_2Cl_2 and dried with sodium sulfate. This was filtered, and the filtrate concentrated. The crude product was purified by a traditional recrystallization from boiling CH_3CN and slowly cooled to yield 50.0 mg (15 %) of a metallic brown solid, mp > 260 °C: ^{1}H NMR [400 MHz, CD_2Cl_2] δ 8.73 (d, 2 H, J = 6.0 Hz), 8.09 (d, 2 H, *J* = 3.2 Hz), 8.03 (s, 2 H), 7.66 (d, 2 H, *J* = 9.2 Hz), 7.59-7.57 (m, 2 H,) 6.85 (d, 2 H, *J* = 9.2 Hz), 3.12 (s, 6 H,); λ_{max} (CH_2Cl_2) 748 nm ($\varepsilon = 7.38 \times 10^4$ $M^{-1}cm^{-1}$), 527 nm ($\varepsilon = 2.66 \times 10^4$ $M^{-1}cm^{-1}$); IR (ATR) 2126.9 cm^{-1} (m, alkyne) 1600.4 cm^{-1} (m, aromatic C=C), 1371.0 cm^{-1} (m, C-N); HRMS (ESI) *m/z* 547.8914 (calcd for $C_{23}H_{18}N_1Se_3^+$: 547.8906); Anal calcd for $C_{23}H_{18}F_6NPSe_3$: C, 40.02; H, 2.63; N, 2.03. Found: C, 39.84; H, 2.80; N 2.16.

Synthesis of N-(6-(dimethylamino)-9-(phenylethynyl)-3H-selenoxanthen-3-ylidene)-N-methylmethanaminium hexafluorophosphate (5a).

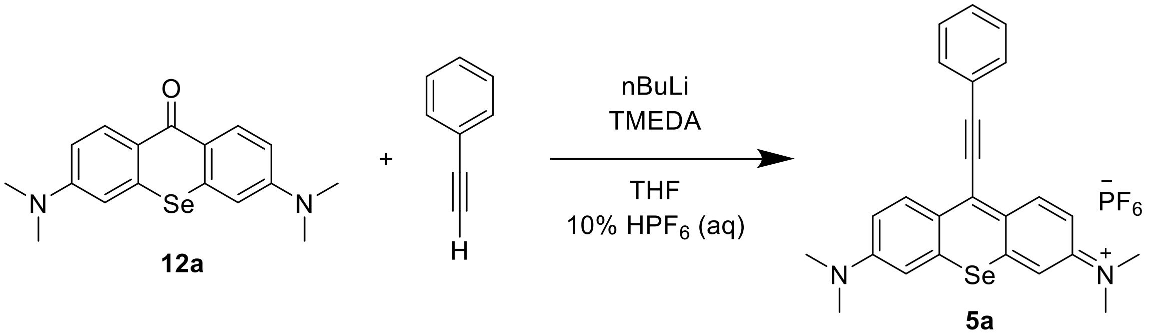

A flame-dried flask fit with a condenser under nitrogen was charged with ethynylbenzene (70.0 µL, 0.637 mmol), tetramethylenediamine (86.6 µL, 0.579 mmol), and THF (5.00 mL) and cooled to -78 °C. To this solution, *n*-butyl lithium (29.1 µL, 0.579 mmol) was added dropwise. In a separate flame-dried flask, 3,6-bis(dimethylamino)-9H-selenoxanthen-9-one (**12a**) (100.0 mg, 0.290 mmol) was dissolved in THF (5.00 mL) and cooled to -78 °C. After 15 min, the contents of the second flask were transferred via cannula to the first flask. The reaction mixture was then allowed to warm to ambient, followed by heating at 50 °C for 15 min. The reaction mixture was cooled to ambient and dumped into a 10 % solution of hexafluorophosphoric acid (30 mL). After 3 h, the mixture was filtered, and washed with water and ether. The collected residue was dissolved in CH_3CN and dried with sodium sulfate. This was filtered, and the filtrate concentrated. The crude product was purified by a two-solvent recrystallization from 10% CH_3CN/Et_2O to yield 45.0 mg (26 %) of a metallic green solid, mp > 230 °C. ^1H NMR [300 MHz, CD_3CN] δ 8.47 (d, 2 H, J = 9.9 Hz), 7.76 (d, 2 H, *J* = 6.9 Hz), 7.55 (m, 3 H, *J* = 7.8 Hz), 7.15 (s, 2 H), 7.02 (d, 2 H, J = 9.0 Hz), 3.15 (s, 12 H); λ_{max} (CH_2Cl_2) 632 nm ($\varepsilon = 7.61 \times 10^4$ $M^{-1}cm^{-1}$); λ_{max} (CH_2Cl_2) 632 nm ($\varepsilon = 7.61 \times 10^4$ $M^{-1}cm^{-1}$); IR (ATR) 2194.3 cm^{-1} (m, alkyne), 1592.4 cm^{-1} (m, aromatic C=C), 1362.6 cm^{-1} (m, C-N); HRMS (ESI) *m/z* 431.1022 (calcd for $C_{25}H_{23}N_2Se^+$: 431.1021).

Synthesis of N-(6-(dimethylamino)-9-((4-methoxyphenyl)ethynyl)-3H-selenoxanthen-3-ylidene)-N-methylmethanaminium hexafluorophosphate (5b).

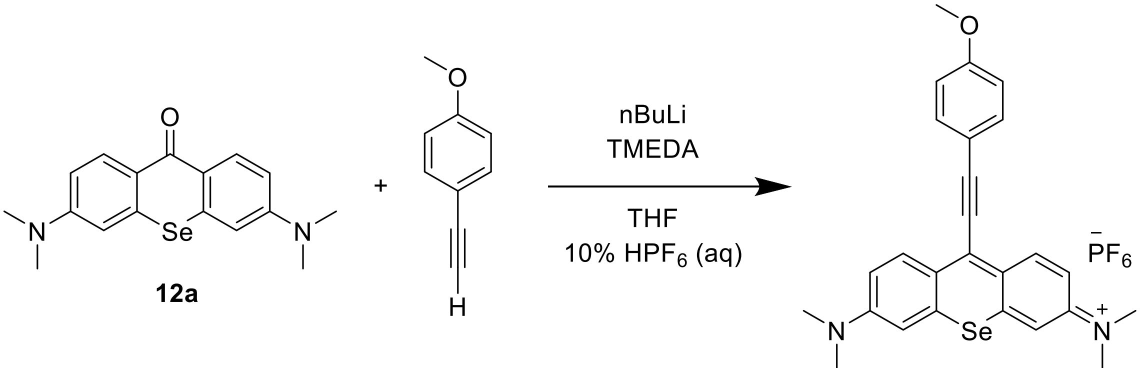

A flame-dried flask fit with a condenser under nitrogen was charged with 1-ethynyl-4-methoxybenzene (82.6 µL, 0.637 mmol), tetramethylenediamine (86.6 µL, 0.579 mmol), and THF (5.00 mL) and cooled to -78 °C. To this solution, *n*-butyl lithium (29.1 µL, 0.579 mmol) was added dropwise. In a separate flame-dried flask, 3,6-bis(dimethylamino)-9H-selenoxanthen-9-one (**12a**) (100.0 mg, 0.290 mmol) was dissolved in THF (5.00 mL) and cooled to -78 °C. After 15 min, the contents of the second flask were transferred via cannula to the first flask. The reaction mixture was then allowed to warm to ambient, followed by heating at 50 °C for 15 min. The reaction mixture was cooled to ambient and dumped into a 10 % solution of hexafluorophosphoric acid (30 mL). After 3 h, the mixture was filtered, and washed with water and ether. The collected residue was dissolved in CH_3CN and dried with sodium sulfate. This was filtered, and the filtrate concentrated. The crude product was purified by a two-solvent recrystallization from 10% CH_3CN/Et_2O to yield 75.0 mg (42 %) of a metallic green solid, mp > 230 °C. ^1H NMR [300 MHz, CD_3CN] δ 8.51 (d, 2 H, J = 9.3 Hz), 7.74 (d, 2 H, *J* = 9.0 Hz), 7.18 (s, 2 H), 7.05 (m, 4 H, J = 6.9 Hz), 3.90 (s, 3 H), 3.17 (s, 12 H); λ_{max} (CH_2Cl_2) 625 nm ($\varepsilon = 9.44 \times 10^4$ $M^{-1}cm^{-1}$); IR (ATR) 2185.6 cm^{-1} (m, alkyne), 1593.8 cm^{-1} (m, aromatic C=C), 1362.5 cm^{-1} (m, C-N); HRMS (ESI) *m/z* 461.1127 (calcd for $C_{26}H_{25}N_2OSe^+$: 461.1127).

Synthesis of N-(6-(dimethylamino)-9-((4-morpholinophenyl)ethynyl)-3H-selenoxanthen-3-ylidene)-N-methylmethanaminium (5c).

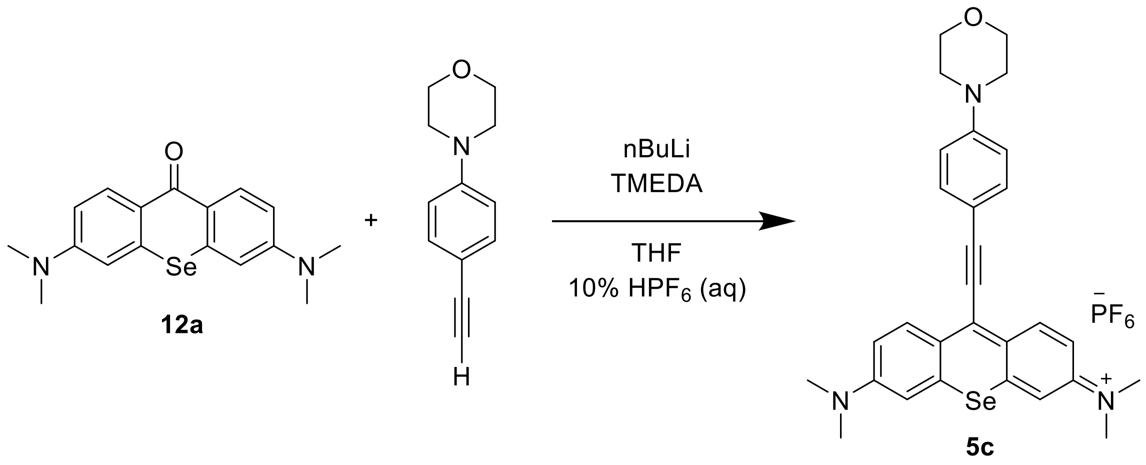

A flame-dried flask fit with a condenser under nitrogen was charged with 4-(4-ethynylphenyl)morpholine (119.3 mg, 0.637 mmol), tetramethylenediamine (86.6 µL, 0.579 mmol), and THF (5.00 mL) and cooled to -78 °C. To this solution, *n*-butyl lithium (29.1 µL, 0.579 mmol) was added dropwise. In a separate flame-dried flask, 3,6-bis(dimethylamino)-9H-selenoxanthen-9-one (**12a**) (100.0 mg, 0.290 mmol) was dissolved in THF (5.00 mL) and cooled to -78 °C. After 15 min, the contents of the second flask were transferred via cannula to the first flask. The reaction mixture was then allowed to warm to ambient, followed by heating at 50 °C for 15 min. The reaction mixture was cooled to ambient and dumped into a 10 % solution of hexafluorophosphoric acid (30 mL). After 3 h, the mixture was filtered, and washed with water and ether. The collected residue was dissolved in CH_3CN and dried with sodium sulfate. This was filtered, and the filtrate concentrated. The crude product was purified by a two-solvent recrystallization from 10% CH_3CN/Et_2O to yield 80.4 mg (42 %) of a metallic green/brown solid, mp > 230 °C. ^1H NMR [300 MHz, CD_3CN] δ 8.49 (d, 2 H, J = 8.4 Hz), 7.64 (s, 2 H), 7.14 (s, 2 H), 7.01 (m, 4 H), 3.82 (t, 4 H), 3.35 (t, 4 H), 3.16 (s, 12 H); λ_{max} (CH_2Cl_2) 621 nm ($\varepsilon = 9.39 \times 10^4$ $M^{-1}cm^{-1}$); IR (ATR) 2173.3 cm^{-1} (m, alkyne), 1588.4 cm^{-1} (m, aromatic C=C), 1360.0 cm^{-1} (m, C-N); HRMS (ESI) *m/z* 516.1552 (calcd for $C_{29}H_{30}N_3OSe^+$: 516.1549).

Synthesis of N-(6-(dimethylamino)-9-((4-(dimethylamino)phenyl)ethynyl)-3H-selenoxanthen-3-ylidene)-N-methylmethanaminium hexafluorophosphate (5d).

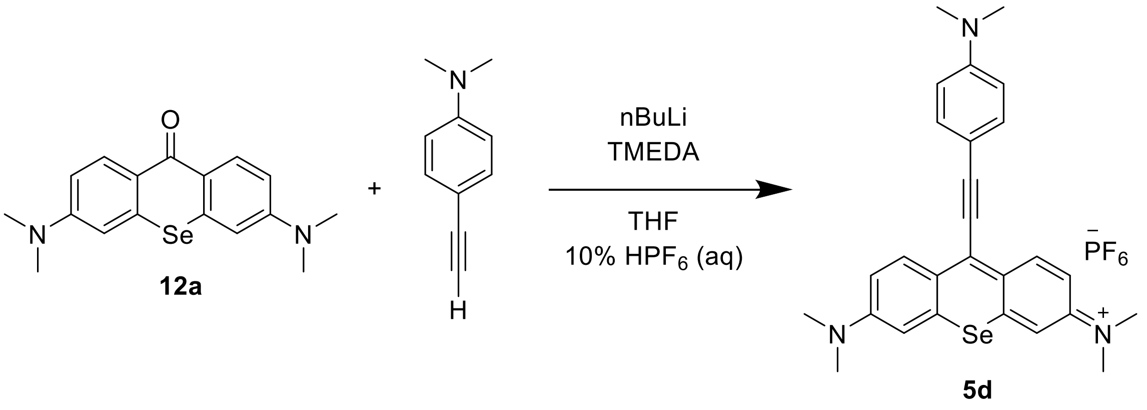

A flame-dried flask fit with a condenser under nitrogen was charged with 4-ethynyl-N,N-dimethylaniline (92.5 mg, 0.637 mmol), tetramethylenediamine (86.6 µL, 0.579 mmol), and THF (5.00 mL) and cooled to -78 °C. To this solution, *n*-butyl lithium (29.1 µL, 0.579 mmol) was added dropwise. In a separate flame-dried flask, 3,6-bis(dimethylamino)-9H-selenoxanthen-9-one (**12a**) (100.0 mg, 0.290 mmol) was dissolved in THF (5.00 mL) and cooled to -78 °C. After 15 min, the contents of the second flask were transferred via cannula to the first flask. The reaction mixture was then allowed to warm to ambient, followed by heating at 50 °C for 15 min. The reaction mixture was cooled to ambient and dumped into a 10 % solution of hexafluorophosphoric acid (30 mL). After 3 h, the mixture was filtered, and washed with water and ether. The collected residue was dissolved in CH_3CN and dried with sodium sulfate. This was filtered, and the filtrate concentrated. The crude product was purified by a two-solvent recrystallization from 10% CH_3CN/Et_2O to yield 63.4 mg (35 %) of a metallic green/brown solid, mp > 230 °C. ^1H NMR [300 MHz, CD_3CN] δ 8.55 (d, 2 H, J = 9.9 Hz), 7.66 (d, 2 H, *J* = 9.0 Hz), 7.17 (s, 2 H), 7.06 (d, 2 H, J = 9.3 Hz), 6.80 (d, 2 H, J = 8.1 Hz), 3.18 (s, 12 H), 3.09 (s, 6 H); λ_{max} (CH_2Cl_2) 613 nm (ε = 1.13 × 10^5 $M^{-1}cm^{-1}$); IR (ATR) 2149.6 cm^{-1} (m, alkyne), 1587.1 cm^{-1} (m, aromatic C=C), 1359.3 cm^{-1} (m, C-N); HRMS (ESI) *m/z* 474.1443 (calcd for $C_{27}H_{28}N_3Se^+$: 474.1443).

Synthesis of N-(9-cyano-6-(dimethylamino)-3H-thioxanthen-3-ylidene)-N-methylmethanaminium hexafluorophosphate (6a).

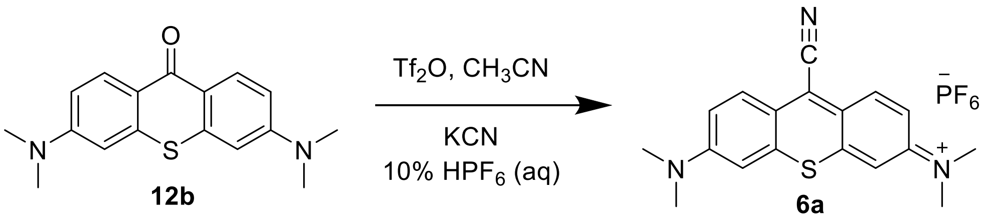

A flame-dried flask under nitrogen was charged with 3,6-bis(dimethylamino)-9H-thioxanthen-9-one (**12b**) (50.0 mg, 0.168 mmol), and CH_3CN (8.00 mL). To this solution, trifluoromethanesulfonic anhydride (40.0 µL, 0.184 mmol) was added dropwise. After 30 min, KCN (54.6 mg, 0.838 mmol) was added. After 3 h, the reaction mixture was dumped into a 10 % solution of hexafluorophosphoric acid (30 mL). After stirring overnight, the mixture was filtered, and washed with water and ether. The collected residue was dissolved in CH_2Cl_2 and dried with sodium sulfate. This was filtered, and the filtrate concentrated. The crude product was purified by a two-solvent recrystallization from 10% CH_2Cl_2/Et_2O to yield 10.0 mg (13 %) of a blue solid, mp > 200 °C. ^1H NMR [300 MHz, CD_2Cl_2] δ 8.27 (d, 2 H, J = 9.6 Hz), 7.24 (d, 2 H, J = 9.3 Hz), 7.13 (s, 2 H), 3.38 (s, 12 H); λ_{max} (CH_2Cl_2) 662 nm ($\varepsilon = 5.28 \times 10^4$ $M^{-1}cm^{-1}$); IR (ATR) 2924.6 cm^{-1} (w, methyl C-H), 1594.1 cm^{-1} (m, aromatic C=C), 1365.7 cm^{-1} (m, C-N); HRMS (ESI) *m/z* 308.1220 (calcd for $C_{18}H_{18}N_3S^+$: 308.1216).

Synthesis of N-(9-cyano-6-(dimethylamino)-3H-selenoxanthen-3-ylidene)-N-methylmethanaminium hexafluorophosphate (6b).

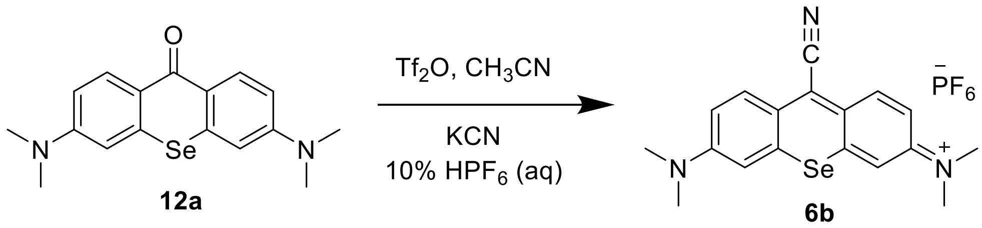

A flame-dried flask under nitrogen was charged with 3,6-bis(dimethylamino)-9H-selenoxanthen-9-one (**12a**) (50.0 mg, 0.145 mmol), and CH_3CN (8.00 mL). To this solution, trifluoromethanesulfonic anhydride (26.7 μL, 0.159 mmol) was added dropwise. After 30 min, KCN (51.9 mg, 0.796 mmol) was added. After 3 h, the reaction mixture was dumped into a 10 % solution of hexafluorophosphoric acid (30 mL). After stirring overnight, the mixture was filtered, and washed with water and ether. The collected residue was dissolved in CH_2Cl_2 and dried with sodium sulfate. This was filtered, and the filtrate concentrated. The crude product was purified by a two-solvent recrystallization from 10% CH_2Cl_2/Et_2O to yield 32.4 mg (45 %) of a blue solid, mp > 200 °C. ^1H NMR [300 MHz, CD_2Cl_2] δ 8.37 (d, 2 H, J = 9.9 Hz), 7.32 (s, 2 H), 7.17 (d, 2 H, J = 9.3 Hz), 3.35 (s, 12 H); λ_{max} (CH_2Cl_2) 669 nm ($\varepsilon = 4.43 \times 10^4$ $M^{-1}cm^{-1}$); IR (ATR) 2926.7 cm^{-1} (w, methyl C-H), 1591.3 cm^{-1} (m, aromatic C=C), 1367.1 cm^{-1} (m, C-N); HRMS (ESI) *m/z* 356.0643 (calcd for $C_{18}H_{18}N_3Se^+$: 356.0660).

Synthesis of 9-cyano-12-(dimethylamino)-1,2,3,5,6,7-hexahydropyrido[3,2,1-ij]thiochromeno[2,3-f]quinolin-4-ium hexafluorophosphate (7a).

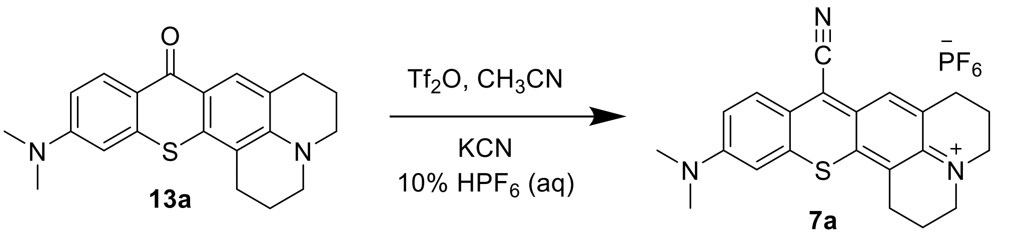

A flame-dried flask under nitrogen was charged with 12-(dimethylamino)-2,3,6,7-tetrahydro-1H,5H,9H-pyrido[3,2,1-ij]thiochromeno[2,3-f]quinolin-9-one (**13a**) (100.0 mg, 0.285 mmol), and CH_3CN (12.00 mL). To this solution, trifluoromethanesulfonic anhydride (52.7 µL, 0.314 mmol) was added dropwise. After 30 min, KCN (92.9 mg, 1.427 mmol) was added. After 3 h, the reaction mixture was dumped into a 10 % solution of hexafluorophosphoric acid (30 mL). After stirring overnight, the mixture was filtered, and washed with water and ether. The collected residue was dissolved in CH_2Cl_2 and dried with sodium sulfate. This was filtered, and the filtrate concentrated. The crude product was purified by a two-solvent recrystallization from 10% CH_2Cl_2/Et_2O to yield 66.9 mg (46 %) of a blue solid, mp > 200 °C. ^{1}H NMR [300 MHz, CD_2Cl_2] δ 8.19 (d, 1 H, J = 9.3 Hz), 7.92 (s, 1 H), 7.16 (d, 1 H, J = 9.3 Hz), 7.09 (s, 1 H), 3.65 (q, 4 H, J = 6.0 Hz), 3.31 (s, 6 H), 2.98 (t, 2 H, J = 6.3 Hz), 2.81 (t, 2 H, J = 6.0 Hz), 2.14 (m, 4 H, J = 5.7 Hz); λ_{max} (CH_2Cl_2) 671 nm (ε = 8.08 × 10^4 $M^{-1}cm^{-1}$); IR (ATR) 2941.2 cm^{-1} (w, methylene C-H), 1592.5 cm^{-1} (m, aromatic C=C), 1359.5 cm^{-1} (m, C-N); HRMS (ESI) *m/z* 360.1537 (calcd for $C_{22}H_{22}N_3S^+$: 360.1529).

Synthesis of 9-cyano-12-(dimethylamino)-1,2,3,5,6,7-hexahydropyrido[3,2,1-ij]selenochromeno[2,3-f]quinolin-4-ium hexafluorophosphate (7b).

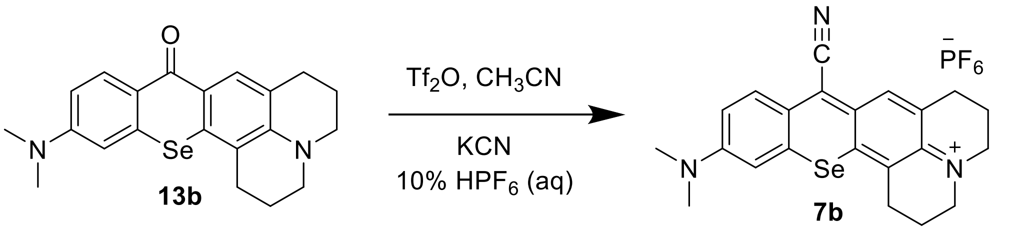

A flame-dried flask under nitrogen was charged with 12-(dimethylamino)-2,3,6,7-tetrahydro-1H,5H,9H-pyrido[3,2,1-ij]selenochromeno[2,3-f]quinolin-9-one (**13b**) (50.0 mg, 0.126 mmol), and CH_3CN (8.00 mL). To this solution, trifluoromethanesulfonic anhydride (23.2 µL, 0.138 mmol) was added dropwise. After 30 min, KCN (50.0 mg, 0.629 mmol) was added. After 3 h, the reaction mixture was dumped into a 10 % solution of hexafluorophosphoric acid (30 mL). After stirring overnight, the mixture was filtered, and washed with water and ether. The collected residue was dissolved in CH_2Cl_2 and dried with sodium sulfate. This was filtered, and the filtrate concentrated. The crude product was purified by a two-solvent recrystallization from 10% CH_2Cl_2/Et_2O to yield 36.0 mg (52 %) of a blue solid, mp > 200 °C. ^{1}H NMR [300 MHz, CD_2Cl_2] δ 8.30 (d, 1 H, J = 9.3 Hz), 8.08 (s, 1 H), 7.26 (s, 1 H), 7.11 (d, 1 H, J = 9.6 Hz), 3.63 (m, 4 H), 3.29 (s, 6 H), 2.97 (t, 2 H, J = 5.7 Hz), 2.71 (t, 2 H, J = 5.7 Hz), 2.16 (m, 4 H, J = 5.4 Hz); λ_{max} (CH_2Cl_2) 675 nm ($\varepsilon = 6.18 \times 10^4$ $M^{-1}cm^{-1}$); IR (ATR) 2943.6 cm^{-1} (w, methylene C-H), 1591.2 cm^{-1} (m, aromatic C=C), 1363.8 cm^{-1} (m, C-N); HRMS (ESI) *m/z* 408.0983 (calcd for $C_{22}H_{22}N_3Se^+$: 408.0973).

Synthesis of N-(9-cyano-6-(julolidyl)-3H-thioxanthen-3-ylidene)-N-methylmethanaminium hexafluorophosphate (8a).

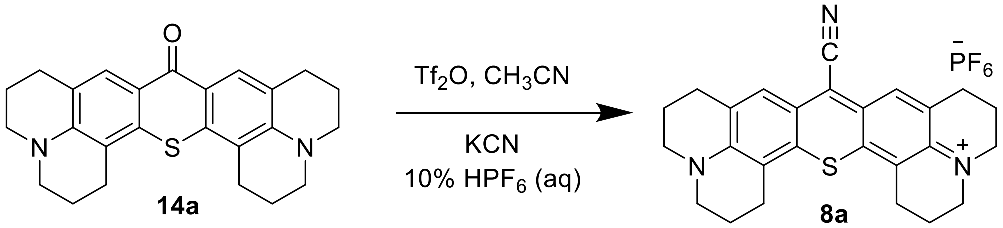

A flame-dried flask under nitrogen was charged with 3,6-bis(julolidyl)-9H-thioxanthen-9-one (**14a**) (50.0 mg, 0.124 mmol), and CH_3CN (8.00 mL). To this solution, trifluoromethanesulfonic anhydride (22.9 µL, 0.137 mmol) was added dropwise. After 30 min, KCN (40.4 mg, 0.621 mmol) was added. After 3 h, the reaction mixture was dumped into a 10 % solution of hexafluorophosphoric acid (30 mL). After stirring overnight, the mixture was filtered, and washed with water and ether. The collected residue was dissolved in CH_2Cl_2 and dried with sodium sulfate. This was filtered, and the filtrate concentrated. The crude product was purified by a two-solvent recrystallization from 10% CH_2Cl_2/Et_2O to yield 29.3 mg (42 %) of a blue solid, mp > 200 °C. ^1H NMR [300 MHz, CD_2Cl_2] δ 7.88 (s, 2H), 3.58 (m, 8 H), 2.92 (m, 8 H), 2.12 (m, 8 H); λ_{max} (CH_2Cl_2) 687 nm ($\varepsilon = 2.11 \times 10^5$ $M^{-1}cm^{-1}$); IR (ATR) 2940.0 cm^{-1} (w, methylene C-H), 1590.7 cm^{-1} (m, aromatic C=C), 1362.1 cm^{-1} (m, C-N); HRMS (ESI) *m/z* 412.1852 (calcd for $C_{26}H_{26}N_3S^+$: 412.1842).

Synthesis of N-(9-cyano-6-(julolidyl)-3H-selenoxanthen-3-ylidene)-N-methylmethanaminium hexafluorophosphate (8b).

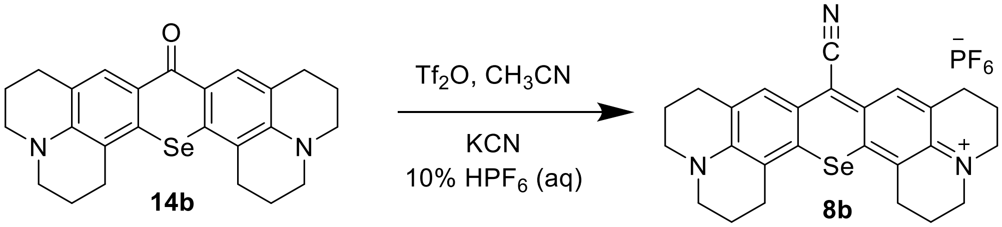

A flame-dried flask under nitrogen was charged with 3,6-bis(julolidyl)-9H-selenoxanthen-9-one (**14b**) (50.0 mg, 0.111 mmol), and CH_3CN (8.00 mL). To this solution, trifluoromethanesulfonic anhydride (20.5 μL, 0.122 mmol) was added dropwise. After 30 min, KCN (36.2 mg, 0.556 mmol) was added. After 3 h, the reaction mixture was dumped into a 10 % solution of hexafluorophosphoric acid (30 mL). After stirring overnight, the mixture was filtered, and washed with water and ether. The collected residue was dissolved in CH_2Cl_2 and dried with sodium sulfate. This was filtered, and the filtrate concentrated. The crude product was purified by a two-solvent recrystallization from 10% CH_2Cl_2/Et_2O to yield 51.6 mg (77 %) of a blue solid, mp > 200 °C. ^1H NMR [300 MHz, CD_2Cl_2] δ 8.01 (s, 2H), 3.56 (m, 8 H), 2.87 (m, 8 H), 2.13 (m, 8 H); λ_{max} (CH_2Cl_2) 692 nm ($\varepsilon = 1.50 \times 10^5$ $M^{-1}cm^{-1}$); IR (ATR) 2943.0 cm^{-1} (w, methylene C-H), 1593.0 cm^{-1} (m, aromatic C=C), 1362.0 cm^{-1} (m, C-N); HRMS (ESI) *m/z* 460.1298 (calcd for $C_{26}H_{26}N_3Se^+$: 460.1286).

Synthesis of 4-(cyano(2,6-di(thiophen-2-yl)-4H-thiopyran-4-ylidene)methyl)-2,6-di(thiophen-2-yl)thiopyrylium hexafluorophosphate (9).

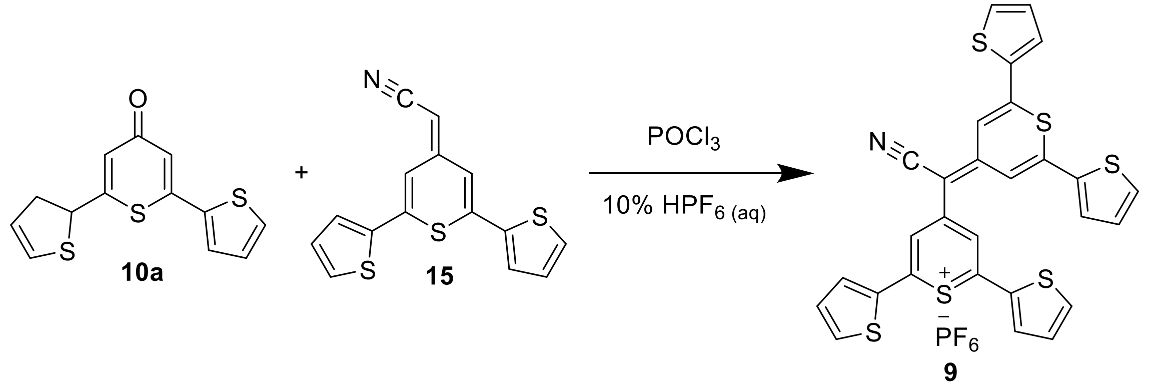

A flame dried flask under nitrogen was charged with 2,6-di(thiophen-2-yl)-4H-thiopyran-4-one (**10a**) (100.0 mg, 0.334 mmol), 2-(2,6-di(thiophen-2-yl)-4H-thiopyran-4-ylidene)acetonitrile (**15**) (101.4 mg, 0.367 mmol) and phosphorus oxychloride (0.33 mL). The reaction mixture was heated at reflux for 3 h. The mixture was then cooled to ambient temperature and poured into a 10 % hexafluorophosphoric acid solution (20 mL). The organic layer was extracted with methylene chloride (6 × 15 mL). The combined organic extracts were dried over sodium sulfate, filtered, and concentrated in vacuo. The residue was crystallized from acetonitrile. The solid was washed with warm portions of CH_3CN. The filtrate was concentrated, and the residual solid was purified by a traditional recrystallization from boiling CH_3CN and slowly cooled to yield 45.1 (20 %) of a copper bronze solid, mp > 260 °C. ^1H NMR [500 MHz, CD_2Cl_2] δ 8.17 (s, 4 H), 7.88-7.87 (m, 8 H), 7.37 (t, 4 H, J = 4.0 Hz); λ_{max} (CH_2Cl_2) = 661 nm (ε 1.3 x 10^5 $M^{-1}cm^{-1}$), 481 nm (ε 4.1 x 10^4 $M^{-1}cm^{-1}$); IR (ATR) 2183.0 cm^{-1} (s, nitrile CN), 1601.6 cm^{-1} (s, aromatic C=C); HRMS (ESI) *m/z* 557.9595 (calcd for $C_{28}H_{16}NS_6^+$: 557.9601). Anal calcd for $C_{28}H_{16}F_6NPS_6$: C, 47.79; H, 2.29; N, 1.99. Found C, 47.99; H, 2.67; N, 2.15.

Supplementary Scheme 1: Synthesis of novel precursor for dye 9.

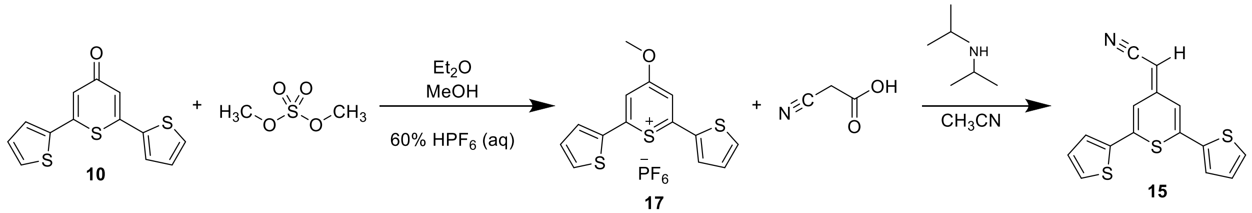

Synthesis of 4-methoxy-2,6-di(thiophen-2-yl)thiopyrylium (17).

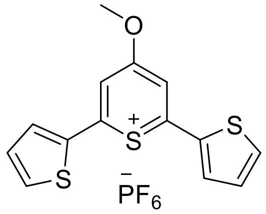

A flame dried flask under nitrogen was charged with 2,6-di(thiophen-2-yl)-4H-thiopyran-4-one (276.4 mg, 1.00 mmol) and dimethyl sulfate (0.54 mL, 5.70 mmol). The reaction mixture was heated at 105 ˚C for 4 h. The mixture was subsequently cooled to ambient temperature. Diethyl ether (20 mL) was added, and the resulting brown residue was collected by vacuum filtration. The brown residue was dissolved in methanol (25 mL). 60 % Hexafluorophosphoric acid was added (10 mL), and the yellow precipitate was collected via vacuum filtration. The yellow solid was dissolved in acetonitrile and dried with sodium sulfate. This was filtered, and concentrated under reduced pressure to afford 225.0 mg (52%) of a yellow solid, mp 139-141 ˚C, ^{1}H NMR [500 MHz, CD_3CN] δ 8.03 (d, 4 H. *J* = 4.0 Hz), 7.91 (s 2 H), 7.38 (t, 2 H, *J* = 4.5 Hz), 7.46 (t, 4 H, J = 4.5 H), 4.34 (s, 3 H); IR (ATR); HRMS (ESI) *m/z* 290.9965 (calcd for $C_{14}H_{11}OS_3$: 290.9967); Anal calcd for $C_{14}H_{11}F_6OPS_3$: C, 38.53; H, 2.54. Found: C, 38.65; H, 2.55.

Synthesis of 2-(2,6-di(thiophen-2-yl)-4H-thiopyran-4-ylidene)acetonitrile (15).

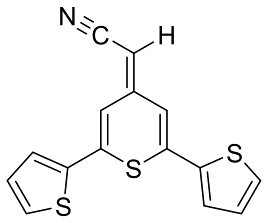

A flame dried flask under nitrogen was charged with 4-methoxy-2,6-di(thiophen-2-yl)thiopyrylium hexafluorophosphate (17) (200.0 mg, 0.458 mmol), cyanoacetic acid (91.6 mg, 1.07 mmol), N,N-diisopropylethylamine (0.19 mL, 1.07 mmol), and anhydrous acetonitrile (1.40 mL). The reaction mixture was heated at reflux for 2 h. The mixture was then cooled to ambient temperature and dumped into water (25 mL). The mixture was then filtered to yield a brown solid, which was dissolved in methylene chloride and dried with magnesium sulfate. This was filtered and concentrated under reduced pressure, and purified by column chromatography over silica gel with a 25% hexanes/CH_2Cl_2 eluent (R_f = 0.90) to yield 92.3 mg (67 %) of a metallic brown solid, mp 113-115 ˚C: ^{1}H NMR [500 MHz, CD_2Cl_2] δ 7.46−7.41 (m, 4 H), 7.27 (s, 1 H), 7.13 (s 2 H), 6.88 (s, 1 H), 4.85 (s. 1 H). HRMS (ESI) *m/z* 299.9970 (calcd for $C_{16}H_9NS_3$: 299.9970). Anal calcd for $C_{16}H_9NS_3$: C, 60.17; H, 3.03; N, 4.68. Found: C, 59.88; H, 2.90; N, 4.68.

Supplementary Scheme 2: Synthesis of novel precursors for dyes 1, 3b, 4b, and 9b.

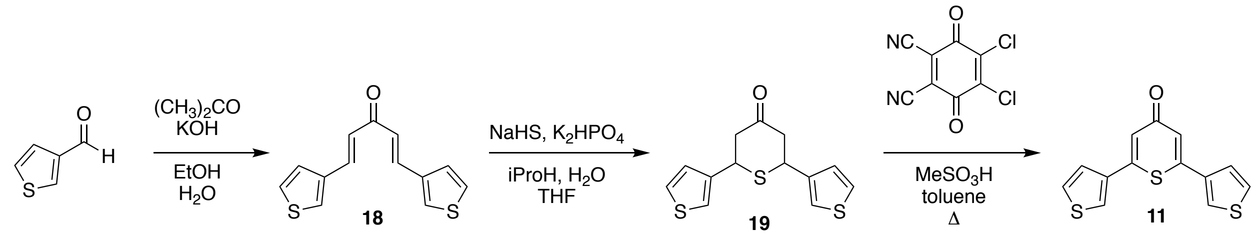

Synthesis of (1*E*,4*E*)-1,5-di(thien-3-yl)penta-1,4-dien-3-one (18).

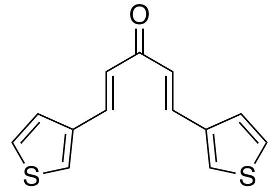

Thiophene-3-carbaldehyde (5.00 g, 44.6 mmol) and acetone (1.29 g, 22.3 mmol) were dissolved in EtOH (40 mL) and KOH (1.25 g, 22.3 mmol) dissolved in H_2O was added slowly. The resulting mixture was stirred for 3h at ambient temperature and was then diluted with H_2O (100 mL). The products were extracted with CH_2Cl_2 (3 × 50 mL) and the combined organic extracts were dried over $MgSO_4$ and concentrated *in vacuo*. The crude product was purified by a two-solvent recrystallization from 10% CH_2Cl_2/hexanes to yield 3.63 g (66%) of a bright yellow, crystalline solid, mp 137-138 °C: ^{1}H NMR [400 MHz, $CDCl_3$] δ 7.71 (d, 2 H, *J* = 16.0 Hz), 7.57 (s, 2 H), 7.37 (s, 4 H), 6.87 (d, 2 H, *J* = 16 Hz); ^{13}C NMR [100 MHz, $CDCl_3$] δ 189.3, 138.1, 136.5, 128.8, 127.0, 125.2, 125.2; HRMS (ESI) *m/z* 247.0244 (calcd for $C_{13}H_{11}OS_2$: 247.0246); Anal calcd for $C_{13}H_{10}OS_2$: C, 63.38; H, 4.09. Found: C, 63.11; H, 4.02.

Synthesis of 2,6-di(thien-3-yl)tetrahydro-4*H*-thiopyran-4-one (19).

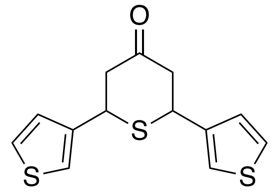

(1*E*,4*E*)-1,5-Di(thiophen-3-yl)penta-1,4-dien-3-one (**18**) (2.00 g, 8.11 mmol) was dissolved in THF (12 mL). To this mixture *i*-PrOH (24 mL) and K_2HPO_4 (2.22 g, 9.74 mmol) dissolved in H_2O (12 mL) were added followed by the addition of NaHS (0.823 g, 8.94 mmol). The resulting mixture was stirred overnight at ambient temperature and was then diluted with H_2O (100 mL). The products were extracted with CH_2Cl_2 (3 × 50 mL). The combined organic extracts were dried over $MgSO_4$ and concentrated *in vacuo*. The crude product was purified by column chromatography over silica gel eluted with 100% CH_2Cl_2 (R_f = 0.65) to yield 2.17 g (95%) of a light-yellow solid, mp 50-55 °C: ^{1}H NMR [400 MHz, $CDCl_3$] δ 7.31 (m, 2 H), 7.22-7.09 (m, 4 H), 4.46 (dd, 1 H, *J* = 3.4, 11.8 Hz), 4.33 (m, 1 H), 3.04 (m, 4 H); ^{13}C NMR [100 MHz, $CDCl_3$] δ 207.9, 207.2, 140.9, 139.9, 127.3, 126.5, 126.4, 126.4, 122.1, 121.8, 50.34, 43.27, 40.16; HRMS (ESI) *m/z* 302.9942 (calcd for $C_{13}H_{12}NaOS_3$: 302.9943); Anal calcd for $C_{13}H_{12}OS_3$: C, 55.68; H, 4.31. Found: C, 55.93; H, 4.38.

Synthesis of 2,6-di(thien-3-yl)-4*H*-thiopyran-4-one (11).

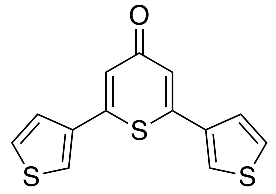

2,6-Di(thien-3-yl)tetrahydro-4*H*-thiopyran-4-one (**19**) (1.50 g, 5.35 mmol) was dissolved in anhydrous toluene (46.8 mL) and placed in a flame-dried flask under nitrogen. 2,3-Dichloro-5,6-dicyano-1,4-benzoquinone (DDQ, 3.04 g, 13.4 mmol) and $MeSO_3H$ (2 drops) were subsequently added. The resulting reaction mixture was heated at reflux for 1.5 h and then cooled to ambient temperature and diluted with saturated aqueous $NaHCO_3$ (50 mL). The product was extracted with CH_2Cl_2 (3 × 50 mL). The combined organic extracts were washed with brine, dried over $MgSO_4$, and concentrated *in vacuo*. The crude product was purified by column chromatography over silica gel eluted with 20% EtOAc/CH_2Cl_2 (R_f = 0.70), and further purified by a two-solvent recrystallization from 10% CH_2Cl_2/hexanes to yield 1.09 g (73%) of a brown solid, mp 130-134 °C: ^{1}H NMR [400 MHz, $CDCl_3$] δ 7.69 (d, 2 H, *J* = 1.2 Hz), 7.45 (m, 2 H), 7.36 (d, 2 H, *J* = 4.8 Hz), 7.16 (s, 2 H); ^{13}C NMR [100 MHz, $CDCl_3$] δ 182.6, 146.5, 136.9, 127.8, 125.6, 125.2, 124.7; HRMS (ESI) *m/z* 276.9809 (calcd for $C_{13}H_9OS_3$: 276.9810); Anal calcd for $C_{13}H_8OS_3$: C, 56.49; H, 2.92. Found: C, 56.21; H, 2.94.

Supplemental References

1. M. A. Bedics, H. Kearns, J. M. Cox, S. Mabbott, F. Ali, N. C. Shand, K. Faulds, J. B. Benedict, D. Graham and M. R. Detty, *Chem. Sci.*, 2015, **6**, 2302-2306.
2. C. H. Chen, G. A. Reynolds and J. A. Van Allan, *J. Org. Chem.*, 1977, **42**, 2777-2778.
3. M.R. Detty, M.; M.A. Bedics, G. Duncan, K.J. Faulds, H. Kearns. Chalcogenopyrylium dyes, compositions comprising same, composite nanoparticles comprising same, and methods of making and using the same. WO2016081813A1, 2016.
4. S. Harmsen, M. A. Bedics, M. A. Wall, R. Huang, M. R. Detty and M. F. Kircher, *Nat. Commun.*, 2015, **6**, 6570
5. P.J. Murphy, *Sci. Synth.* **2003,** *14*, 855-879.
6. D.J. Del Valle, D.J. Donnelly, J.J. Holt, M.R. Detty, *Organometallics*. **2005**, *24*, 3807-3810.
7. J.J. Holt, B.D. Calitree, V. Josiah, M.K. Gannon, II; M.R. Detty, *J. Org. Chem.* **2007**, *72*, 2690-2693.
8. M. W. Kryman, G. A. Schamerhorn, J. E. Hill, B. D. Calitree, K. S. Davies, M. K. Linder, T. Y. Ohulchanskyy and M. R. Detty, *Organometallics*, 2014, **33**, 2628-2640.
